# A Conserved NS3 Surface Patch Orchestrates NS2 Protease Stimulation, NS5A Hyperphosphorylation and HCV Genome Replication

**DOI:** 10.1371/journal.ppat.1004736

**Published:** 2015-03-16

**Authors:** Olaf Isken, Ulrike Langerwisch, Vlastimil Jirasko, Dirk Rehders, Lars Redecke, Harish Ramanathan, Brett D. Lindenbach, Ralf Bartenschlager, Norbert Tautz

**Affiliations:** 1 Institute of Virology and Cell Biology, University of Lübeck, Germany; 2 Department of Molecular Virology, University of Heidelberg, Heidelberg, Germany; 3 Joint Laboratory for Structural Biology of Infection and Inflammation of the University of Hamburg and the University of Lübeck, DESY, Hamburg, Germany; 4 Department of Microbial Pathogenesis, Yale University, New Haven, Connecticut, United States of America; The Scripps Research Institute, UNITED STATES

## Abstract

Hepatitis C virus (HCV) infection is a leading cause of liver disease worldwide. The HCV RNA genome is translated into a single polyprotein. Most of the cleavage sites in the non-structural (NS) polyprotein region are processed by the NS3/NS4A serine protease. The vital NS2-NS3 cleavage is catalyzed by the NS2 autoprotease. For efficient processing at the NS2/NS3 site, the NS2 cysteine protease depends on the NS3 serine protease domain. Despite its importance for the viral life cycle, the molecular details of the NS2 autoprotease activation by NS3 are poorly understood. Here, we report the identification of a conserved hydrophobic NS3 surface patch that is essential for NS2 protease activation. One residue within this surface region is also critical for RNA replication and NS5A hyperphosphorylation, two processes known to depend on functional replicase assembly. This dual function of the NS3 surface patch prompted us to reinvestigate the impact of the NS2-NS3 cleavage on NS5A hyperphosphorylation. Interestingly, NS2-NS3 cleavage turned out to be a prerequisite for NS5A hyperphosphorylation, indicating that this cleavage has to occur prior to replicase assembly. Based on our data, we propose a sequential cascade of molecular events: in uncleaved NS2-NS3, the hydrophobic NS3 surface patch promotes NS2 protease stimulation; upon NS2-NS3 cleavage, this surface region becomes available for functional replicase assembly. This model explains why efficient NS2-3 cleavage is pivotal for HCV RNA replication. According to our model, the hydrophobic surface patch on NS3 represents a module critically involved in the temporal coordination of HCV replicase assembly.

## Author Summary

Hepatitis C virus (HCV) replicates its genome in close association to cellular membranes which serve as assembly site of multi-subunit replication complexes. The process of replication complex maturation must be properly controlled to prevent the non-functional maturation/assembly of these complexes. In this process, the temporal regulation of viral polyprotein processing often plays a pivotal role as exemplified by the strict requirement for NS2-NS3 cleavage for HCV genome replication. We demonstrate here that a conserved hydrophobic NS3 surface patch activates the NS2 protease to stimulate NS2-NS3 cleavage. By dissecting the role of these NS3 surface residues in viral RNA replication, we show that one of these NS3 residues is also a critical determinant for HCV genome replication by negatively regulating NS5A hyperphosphorylation. Surprisingly, further experiments revealed that the NS2-NS3 cleavage is a prerequisite for NS5A hyperphosphorylation. To fulfill the requirements for gradual assembly into functional replication complexes, an ordered cascade of molecular events takes place: in uncleaved NS2-NS3, the hydrophobic NS3 surface patch promotes NS2 protease stimulation; upon NS2-NS3 cleavage, this surface region becomes available for functional replicase assembly. As a consequence, the hydrophobic surface patch on free NS3 can promote NS5A hyperphosphorylation as an indication of functional replicase assembly.

## Introduction

Hepatitis C virus (HCV) is a single-stranded positive-sense RNA virus belonging to the family *Flaviviridae* and is with 170 million infected individuals worldwide an important cause of chronic liver disease [1]. The positive sense RNA genome contains a 5’-untranslated region (UTR), a single open reading frame (ORF) that encodes both structural as well as non-structural (NS) viral proteins and a 3’ UTR. Cap-independent translation of the viral genome yields a single polyprotein that is co- and posttranslationally processed into the individual proteins by host signal peptidases and two viral proteases NS2-NS3 and NS3-4A. The host signal peptidases cleave at the junctions of Core/E1, E1/E2, E2/p7 and p7/NS2 [2–4]. The NS3/NS4A serine protease complex mediates the cleavages of the non-structural proteins NS3-NS5B [5,6]. The chymotrypsin-like serine protease domain residing in the N- terminal 180 amino acids of NS3 requires NS4A as a cofactor for full activity [5,7,8]. NS3 harbors downstream of the protease domain ATPase and helicase activities [9].

The NS2 protein (217 amino acids, aa) is membrane-associated via its N-terminal domain that consists of three putative transmembrane segments with a perinuclear ER localization [10,11]. The C-terminal protease domain (aa 94–217) resides on the cytoplasmic face of the ER membrane [10,12] and, with the N-terminal domain of NS3, forms the NS2-NS3 autoprotease that catalyzes the cleavage at the NS2/NS3 site [7,13,14]. The putative catalytic triad of the NS2-NS3 protease resides entirely in NS2 and autocleavage at the NS2/NS3 junction is independent of the NS3 serine protease activity [7,15,16]. The NS2 protease domain is highly conserved among HCV genotypes and its crystal structure indicates that a dimer forms a composite active site with a catalytic triad analogous to those of cysteine proteases [16]. Recently, NS2, followed by only two residues of NS3, has been shown to be a *bona fide* protease exhibiting low-level intrinsic protease activity. This NS2 protease activity is stimulated by the NS3 serine protease domain (residues 1–180) defining this domain as stimulatory cofactor for NS2 [17]. Cleavage between NS2 and NS3 is essential for RNA genome replication of the full-length virus and subgenomic NS2-NS5B replicons but not for NS3/4A serine protease activity [18–20]. Furthermore, the NS2 protein, but not its proteolytic activity, is required for the production of infectious virus [21–29]. The crystal structure of the NS2 protease domain represents the post-cleavage conformation, which likely differs from the one of the NS2-NS3 precursor [16]. Due to the lack of the NS2-NS3 structure, little is known about the NS2-NS3 cleavage mechanism and its regulation by NS3. Mechanistically, it was proposed that conserved surface areas of NS2 and NS3 may interact to contribute to a functional catalytic NS2-NS3 environment and correct positioning of the scissile bond [16].

To identify molecular features that are critical for NS2 protease stimulation by NS3, we conducted a comprehensive mutagenesis screen of the entire NS3 protease domain. We identified a conserved hydrophobic NS3 surface patch that is essential for efficient NS2 protease activation in the context of uncleaved NS2-NS3 and demonstrate that NS2 protease stimulation mainly depends on hydrophobic protein-protein interactions. Furthermore, mutational analysis of this NS3 surface patch revealed that this area is also pivotal for viral RNA replication and NS5A hyperphosphorylation. Based on these findings we propose a sequential cascade of molecular events where the hydrophobic NS3 surface patch orchestrates NS2 protease stimulation, NS5A hyperphosphorylation and viral RNA replication. Moreover, this study defines an unexpected effect of the NS2-NS3 cleavage and offers a molecular understanding why efficient NS2-NS3 cleavage and the generation of free NS3 are essential for viral RNA replication.

## Results

### Identification of amino acids in the NS3 protease domain required for the activation of the NS2 protease by alanine scanning mutagenesis

**Fig 1:**
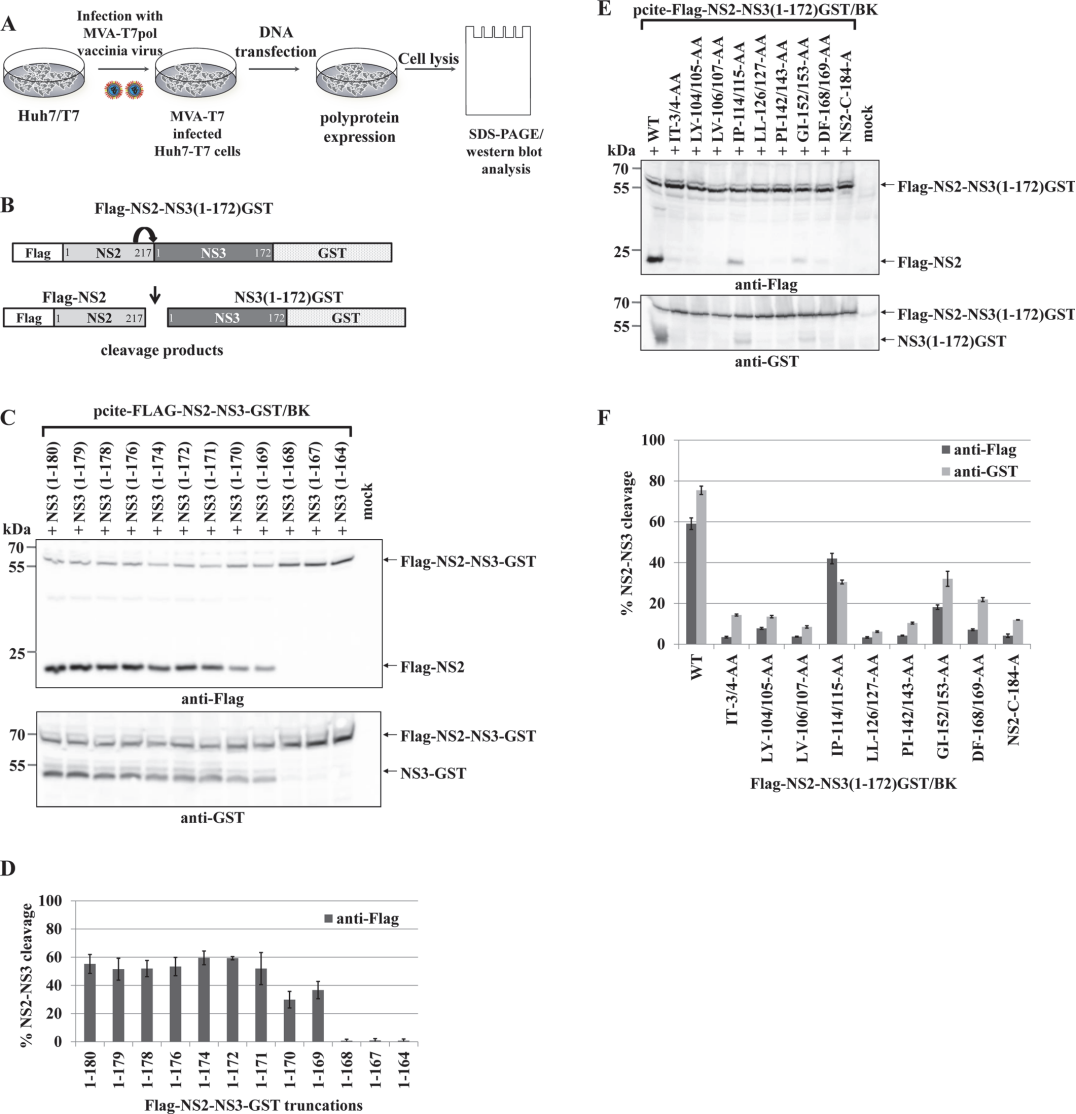
Di-alanine scanning mutagenesis of the NS3 protease domain identified amino acids important for the NS2-protease activation by NS3. **(A)** The workflow to identify NS3 mutations that inhibit NS2-NS3 cleavage. **(B)** Scheme of the HCV NS2-NS3 cleavage assay. NS2-mediated cleavage of expressed FLAG-NS2-NS3(1–172)GST/BK polyprotein fragments results in the generation of FLAG-NS2 and NS3(1–172)GST cleavage products. **(C)** Effect of NS2-NS3 truncations on NS2-NS3 autoprocessing. Plasmids with the indicated NS3 C-terminal truncations were expressed and NS2-NS3 cleavage was detected by Western blotting. NS3(1–180) refers to FLAG-NS2-NS3(1–180)GST/BK. Mock indicates cells transfected with a pcite2a vector control. The positions of FLAG-NS2-NS3-GST, FLAG-NS2 and NS3-GST proteins are indicated by arrows. **(D)** Levels of NS2-NS3 cleavage were quantified from Western blots. **(E)** Inhibition of NS2-NS3 cleavage by selected alanine mutations in NS3. Plasmids encoding FLAG-NS2-NS3(1–172)GST with the indicated NS3 mutations were expressed and NS2-NS3 cleavage was detected by Western blotting. WT refers to wild type FLAG-NS2-NS3(1–172), and NS2-C184-A refers to a plasmid expressing NS2-NS3 polyprotein with a NS2-protease active site mutation. Mock indicates cells transfected with a vector control. The positions of the FLAG-NS2-NS3(1–172)GST, NS3(1–172)GST, and FLAG-NS2 proteins are indicated by arrows. **(F)** Signals of Flag-NS2-NS3GST and NS3GST derivatives were quantified by ImageJ software from three Western blots to calculate the percentage of NS2-NS3 cleavage.

The NS2 protease has a low-level intrinsic ability to cleave the NS2-3 precursor protein and this function is strongly enhanced by the NS3 protease domain (aa 1–180) [17,30]. The NS2-NS3 cleavage can be analyzed in a cell-based assay by expressing HCV NS2-NS3 polyprotein fragments from T7 promoter of pcite-FLAG-NS2-NS3(1–172)GST plasmid in T7 polymerase producing Huh-7/T7 cells additionally boosted by the MVA-T7pol vaccinia virus system followed by Western blot analysis (Fig. 1A and 1B).

To investigate the mechanism of NS2-NS3 cleavage, we aimed at the identification of residues in the NS3 protease domain that are critical for NS2 stimulation. Accordingly, we established an experimental set-up that retains robust NS2-NS3 cleavage but yet is sensitive to perturbations of NS2 activation when mutating residues in NS3. To this end, analysis of C-terminal truncations of the NS3 protease domain revealed that NS3 residues 1–169 were able to detectably activate NS2-NS3 cleavage. Furthermore, a NS3 protease domain consisting of aa 1–172 was found to be sufficient to stimulate NS2-NS3 cleavage to a level comparable with NS2-NS3(1–180) (Fig. 1C and 1D). Therefore, we used pcite-FLAG-NS2-NS3(1–172)GST as the basis for our di-alanine scanning mutagenesis of the entire (genotype 1b)-derived NS3 protease domain and determined the impact of these di-alanine NS3 mutations on the NS2 activation by measuring the NS2-NS3 cleavage by Western blotting (Fig. 1A and 1B).

Among all tested NS3 di-alanine mutants we identified 8 candidates that either strongly interfere with (IP114/115AA and GI152/153AA) or inhibit (IT3/4AA, LY104/105AA, LV106/107AA, LL126/127AA, PL142/143AA and DF168/169AA) the NS2-NS3 cleavage (Fig. 1E and 1F). To rule out that these NS3 mutations affect NS3 protein function(s) not related to the NS2-activation, we introduced the inhibitory mutations into pcite-NS3(1–172)GST/BK and determined their NS3 serine protease activity in a *trans*-cleavage assay in the presence of the NS4A cofactor with a NS4B/NS5A serine protease substrate (S1A Fig.). While the NS3 mutations IT3/4AA and IP114/115AA exhibit serine protease activity comparable to wild type NS3 in this *trans*-cleavage assay, mutations LY104/105AA and LL126/127AA reduced NS3 serine protease activity but still allowed for detectable cleavage of the NS4B/NS5A substrate (S1B and S1C Fig.). In contrast, the NS3 double mutations LV106/107AA, PL142/143AA, GI152/153AA and DF168/169AA did not display detectable NS3 serine protease activity in this assay (S1B and S1C Fig.). Mapping of these NS3 residues on a NS3 structure revealed that residues LV106/107, PL142/143, GI152/153 and DF168/169 are directed towards the center of the NS3 protein suggesting that changing these amino acids pairwise to alanine might affect proper NS3 protein folding (S2 Fig.). Based on these observations only the NS3 mutations IT3/4AA, LY104/105AA, IP114/115AA and LL126/127AA block the NS2 protease activating function of NS3 but still allow for NS3/NS4A serine protease activity. Accordingly, NS3 mutations LV106/107AA, PI142/143AA, GI152/153AA and DF168/169AA were not further analyzed.

### A hydrophobic surface patch in the NS3 protease domain is the main determinant for the NS2 protease stimulation by NS3

**Fig 2:**
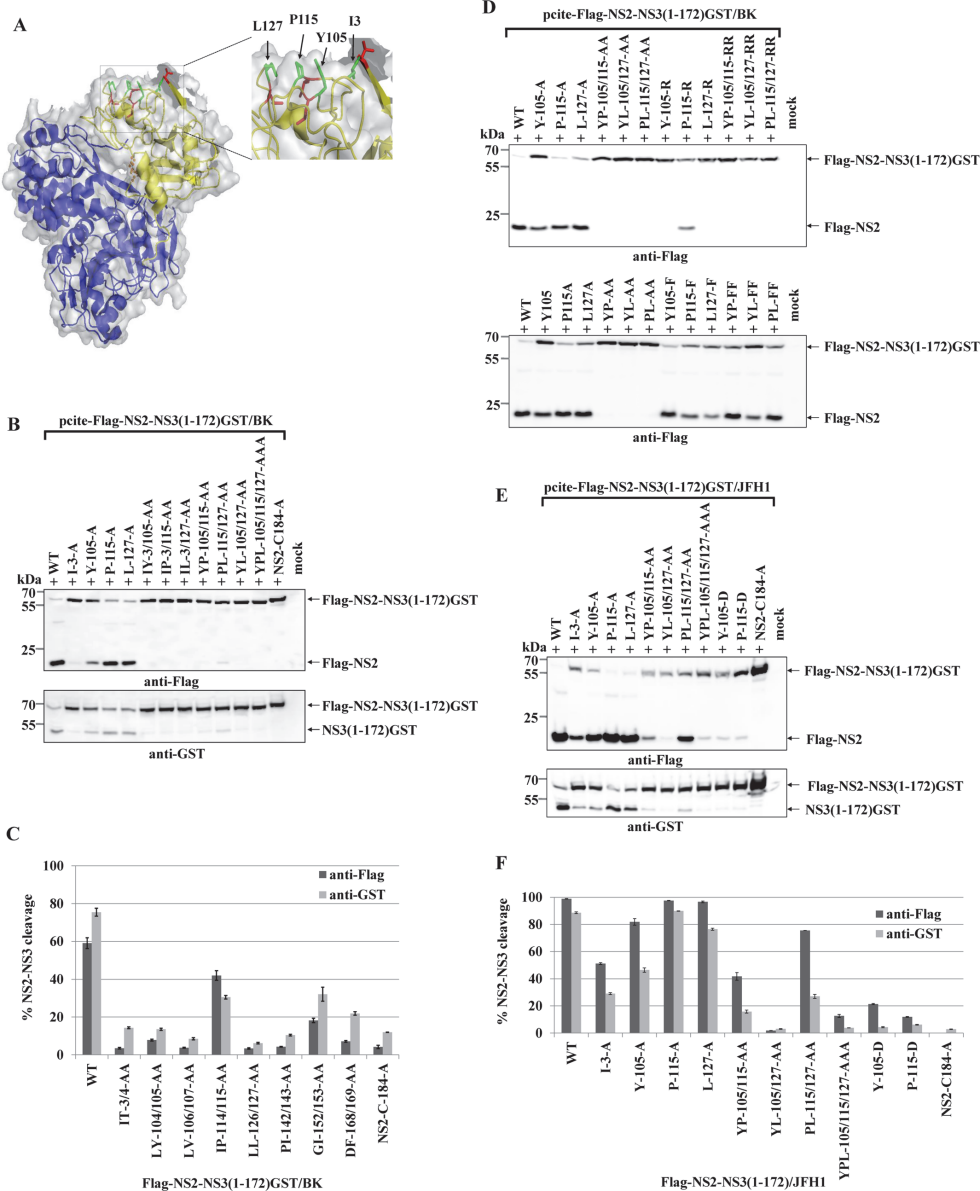
A conserved hydrophobic NS3 surface patch consisting of residues Y105, P115 and L127 is important for NS2-activation by NS3. **(A)** Location of the residues I3, Y105, P115 and L127 in the NS3 structure of the genotype 1b. The surface residues are shown in green stick representation. The residues T4, L104, I114 and L126 are depicted in red stick representation. The overall NS3 structure is shown in grey surface representation. Carbon and backbone ribbon are colored in yellow for the protease domain and blue for the helicase domain, respectively. An enlargement of the NS3 surface patch consisting of I3, Y105, P115 and L127 is shown on the right. The figure was generated using Pymol version 1.10 and the coordinates of PDB code 1CU1 [30]. **(B)** Effect of alanine substitutions in NS3 of HCV strain BK on NS2-NS3 cleavage efficiency. Plasmids expressing either wild type (WT) or mutant FLAG-NS2-NS3(1–172)GST/BK were transfected into Huh7/T7 cells and the cleavage activity was analyzed by Western blot analysis. **(C)** Quantification of NS2-NS3 cleavage from Western blots related to Fig. 2B using ImageJ software. **(D)** The hydrophobic character of the NS3 surface area is important for the NS2 protease stimulation by NS3. The cleavage efficiencies of the indicated BK NS2-NS3 polyprotein fragments carrying either charged (arginine) or hydrophobic (phenylalanine) NS3 amino acid substitutions were determined by Western blot analysis. Quantification of these Western blots is presented in S3 Fig. **(E)** The importance of hydrophobic NS3 surface residues Y105, P115 and L127 for NS2-activation by NS3 is conserved in HCV genotype 2a (JFH1). Plasmids expressing either wild type (WT) or mutant FLAG-NS2-NS3(1–172)GST/JFH1 were transfected into Huh-7/T7 cells and NS2-NS3 cleavage was analyzed by Western blot analysis. Blots shown are representative from three different experiments. **(F)** Quantification of NS2-NS3 cleavage from Western blots related to Fig. 2E.

Since activation of NS2 by NS3 should require interaction of NS2 with NS3, we hypothesized that the amino acids involved, should be localized on the NS3 surface. Mapping of NS3 mutations that selectively blocked NS2 protease activation onto the NS3 crystal structure [31] revealed that I3, Y105, P115 and L127 constitute a continuous hydrophobic surface patch, while the residues T4, L104, I114 and L126 are directed more towards the protein core (Fig. 2A).

**Fig 3:**
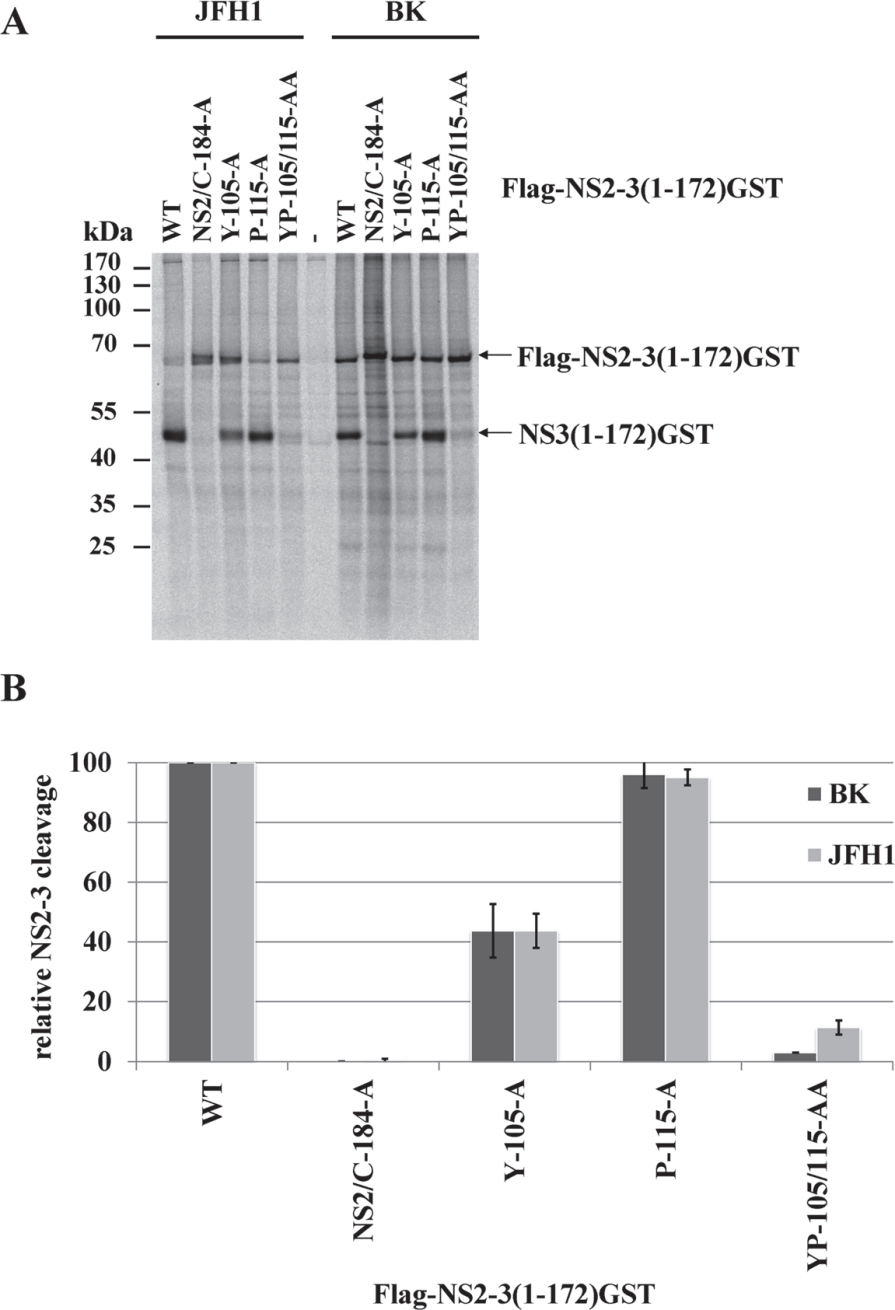
Mutational analysis of the NS3-mediated NS2 protease stimulation demonstrates that this process is conserved among different HCV genotypes. **(A)** Transient expression of Flag-NS2-3(1–172)GST derivatives from genotype 1b (BK) and genotype 2a (JFH1). After transient expression in the presence of [^35^S] methionine/cysteine by use of the T7 vaccinia virus system, the cell lysates were subjected to a radioimmunoprecipitation analysis using an anti-GST antibody. The transfected pcite Flag-NS2-NS3(1–172)GST plasmids encoding NS2-3 polyprotein fragments of genotype 1b (BK) or genotype 2a (JFH1) are indicated. WT, wild type; NS2/C184A, NS2-protease inactive mutant; mock, lysates of MVA-T7pol-infected cells were used for RIP assay. The positions of Flag-NS2-NS3(1–172)GST and NS3(1–172)GST are indicated by arrowheads. The positions of the mass standards are shown on the left. **(B)** Quantification of two independent RIP assays by phosphoimaging.

To investigate if this surface area is critical for the NS2 protease stimulation by NS3, we mutated these residues individually as well as simultaneously and analyzed their impact on NS2-NS3 cleavage. While the individual NS3 mutations Y105A, P115A and L127A allowed for NS2-NS3 cleavage to different degrees, their combinations inhibited NS2-NS3 processing, indicating that the identified hydrophobic patch is indeed pivotal for the NS3-mediated NS2 protease stimulation (Fig. 2B). Since the I3A mutation is located in the proximity of the NS2/NS3 site and thus might act as a cleavage site mutant we did not focus on this residue. In order to evaluate if the hydrophobic character or the amino acid identity are critical for NS2 activation, we mutated the surface amino acids Y105, P115 and L127 individually and simultaneously to either phenylalanine or arginine. While all single or double phenylalanine exchanges were functionally tolerated to various degrees (Fig. 2D), the introduction of a single charged amino acid at position 105 (Y105R) or 127 (L127R) strongly inhibited NS2 protease activation (Fig. 2D and S3 Fig.). In contrast, a single proline-to-arginine exchange at position 115 (P115R) had only a moderate effect on NS2-NS3 cleavage indicating more flexibility at this position provided that the surrounding area remains hydrophobic (Fig. 2D and S3 Fig.). These results revealed that the hydrophobicity of the amino acid side chains rather than their identities determine the function of this NS3 surface area for NS2 protease stimulation. The fact that the NS3 residues Y105, P115 and L127 are conserved among all HCV genotypes suggested that this hydrophobic patch and its role in the NS3-mediated NS2 activation represents a conserved feature in the HCV life cycle. To confirm this assumption, we introduced the mutations into pcite-FLAG-NS2-NS3(1–172)GST/JFH1 that is based on genotype 2a (JFH1) NS2-NS3 sequence and determined the extent of the NS2-NS3 cleavage. Overall, the efficiency of the NS2-NS3 cleavage in the presence of the minimal NS3-cofactor domain (aa 1–172) for JFH1 appears to be increased when compared to the genotype 1b (compare Fig. 2B and 2D). As observed for genotype 1b, individual exchanges of P115A and L127A had a minor influence on the NS2-NS3 cleavage efficiency in this genotype 2a context with Y105A displaying a moderate effect (Fig. 2F). In contrast, mutating two (YP105/115AA, YL105/127AA and to a lesser extent PL115/127AA) or all three (YPL105/115/127AAA) of the conserved surface residues simultaneously to alanine, strongly (YP105/115AA, YL105/127AA and YPL105/115/127AAA) or moderately (PL115/127AA) reduced the NS2 activation by NS3 (Fig. 2D). To further confirm this observation, the Flag-NS2-NS3(1–172)GST derivatives with either single (Y105A and P115A) or double alanine substitutions (YP105/115AA) in the hydrophobic patch of two different HCV genotypes, genotype 1b (BK) and genotype 2a (JFH1), were analyzed by a RIP assay. The WT and the NS2/C184A derivatives of both genotypes served as positive and negative controls, respectively. As shown in Fig. 3, the impact of the NS3 mutations on NS2-NS3 cleavage is in agreement with our Western blot results (compare Fig. 3B with Fig. 2C and 2F). Furthermore, the corresponding mutations in both genotypes have a similar impact on the NS2-NS3 cleavage when compared to their respective WT derivate (Fig. 3B). This demonstrates that the mechanism of the NS3-mediated NS2 protease stimulation is conserved among different genotypes.

### The NS3 L127 residue of the hydrophobic surface patch plays an important role in regulating NS5A hyperphosphorylation and viral RNA replication

**Fig 4:**
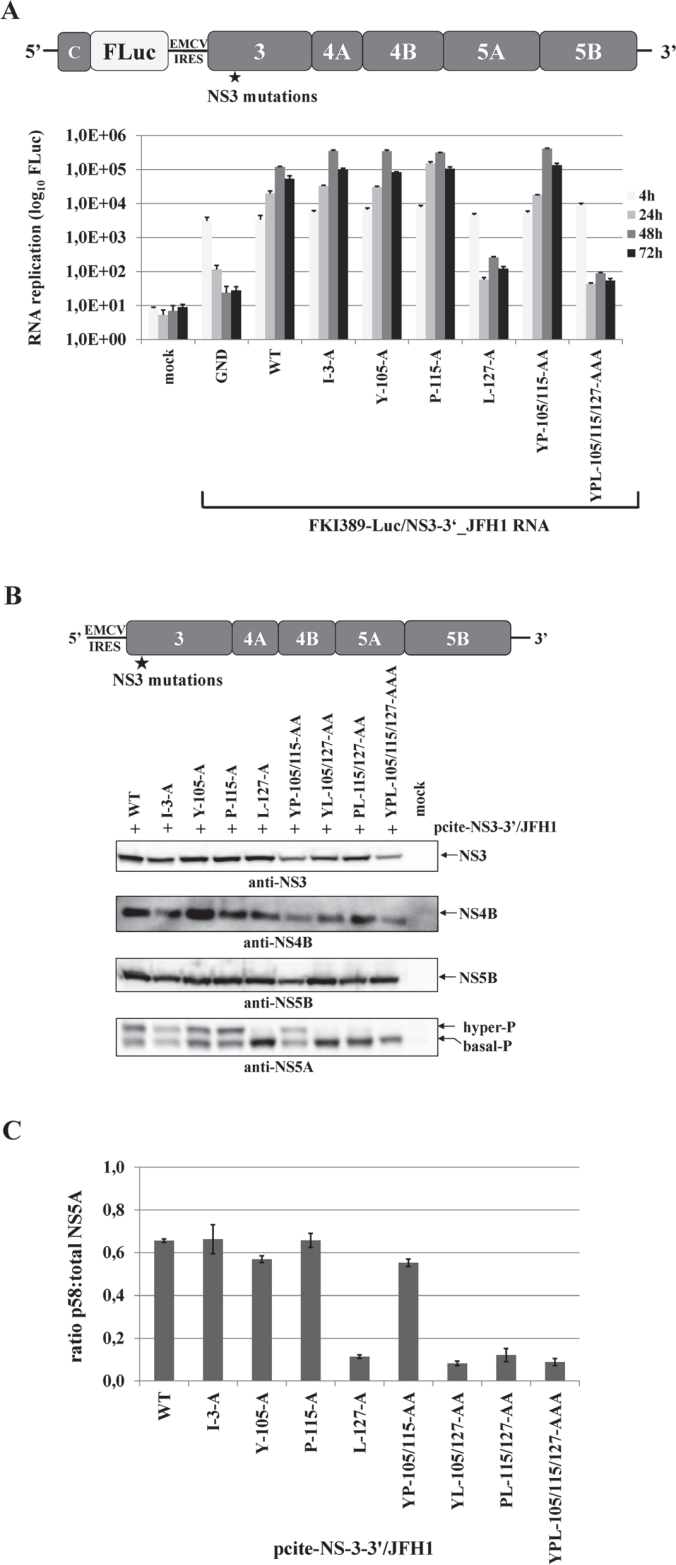
Mutational analysis of the hydrophobic NS3 surface area in the context of a HCV genotype 2a NS3-5B replicon. **(A)** Top, schematic representation of the HCV genotype 2a NS3-5B replicon. Bottom, NS3 mutations were engineered into pFKI389-Luc/NS3-3’_JFH1 (WT), *in vitro* transcribed RNA was electroporated into Huh7 Lunet cells, and luciferase activity was measured at 4, 24, 48 and 72 h post electroporation (pe). Mean values of three independent experiments are shown. Error bars indicate standard deviations. **(B)** Top, schematic of the expression construct pcite-NS3-3’/JFH1. This plasmid encodes the NS3 to NS5B sequence of the HCV genotype 2a (JFH1) as indicated. NS3 mutations were introduced into pcite-NS3-3’/JFH1 and plasmids were transfected into Huh-7/T7cells infected with MVA-T7pol vaccinia virus. Bottom, effect of the NS3 mutations on NS3-NS5B polyprotein processing and NS5A hyperphosphorylation. Cell lysates were prepared 20 h post transfection (pt) and analyzed by Western blotting with antibodies directed against HCV NS3, NS4B, NS5A and NS5B. The position of hyper and basally phosphorylated NS5A is indicated on the right. **(C)** Western blot signals of NS5A p56 and p58 forms of three Western blots were quantified by ImageJ software and the ratio of hyperphosphorylation (p58) to total NS5A was calculated. WT, wild type; GND, polymerase inactive mutant; mock, cells transfected with vector control.

Assuming that the hydrophobic patch on the NS3 surface functions only in NS3-cofactor mediated NS2-stimulation, surface mutations in the context of a NS3-5B/JFH1 replicon should not affect polyprotein processing or RNA replication. To test this hypothesis, we introduced the NS3 mutations into the luciferase-expressing genotype 2a reporter replicon pFKI389-Luc/NS3-3’ and determined the RNA replication kinetics of the mutant RNAs relative to the wild type (WT) and non-replicative (GND) RNAs. Besides the four single mutations (I3A, Y105A, P115A and L127A), two simultaneous mutations (YP105/115AA and YPL105/115/127AAA) were analyzed. The majority of the single alanine mutations (I3A, Y105A or P115A) as well as the combination of Y105 and P115 mutations (YP105/115AA) had no detectable effect on RNA replication, indicating that the I3, Y105 and P115 surface exchanges do not inhibit any of the NS3 functions required for RNA replication in the NS3-NS5B/JFH1 replicon context (Fig. 4A). In contrast, the L127A exchange resulted in a strongly reduced RNA replication level when present either alone or in combination with Y105A or P115A mutations in the NS3-5B/JFH1 replicon, suggesting that this residue plays a critical role in HCV genome replication (Fig. 4A). To characterize the replication phenotype of L127A in more detail, we first determined if this mutation is affecting the NS3-4A serine protease activity. Accordingly, we introduced the entire set of NS3 mutations into a pcite-NS3-3’/JFH1 plasmid which allows the replication-independent expression of the NS3-5B polyprotein by using the MVA-T7pol vaccinia virus system. Western blot analysis confirmed that all NS3 surface mutations (including L127A) had no detectable effects on NS3 stability or NS3-NS5B polyprotein processing in this system (Fig. 4B). In contrast, the analysis of the NS5A phospho-form distribution led to an intriguing observation regarding levels of hyperphosphorylated NS5A. While the NS3 mutations I3A, Y105A, P115A and YP105/115AA exhibited NS5A hyperphosphorylation levels comparable to wild type, the L127A mutation individually or in combination with Y105A, P115A or YP105/115AA showed a strong reduction in NS5A hyperphosphorylation (Fig. 4B). Strikingly, the L127A-replication phenotype correlated with the significant reduction in the ratio of hyper- to basally phosphorylated NS5A (Fig. 4A and 4C). However, we cannot rule out that L127A also inhibits other NS3 function(s), not linked to its serine protease activity, which may also contribute to the negative replication phenotype.

### NS3 mutations defective in NS2 protease stimulation block RNA replication in a NS2-5B replicon system

**Fig 5:**
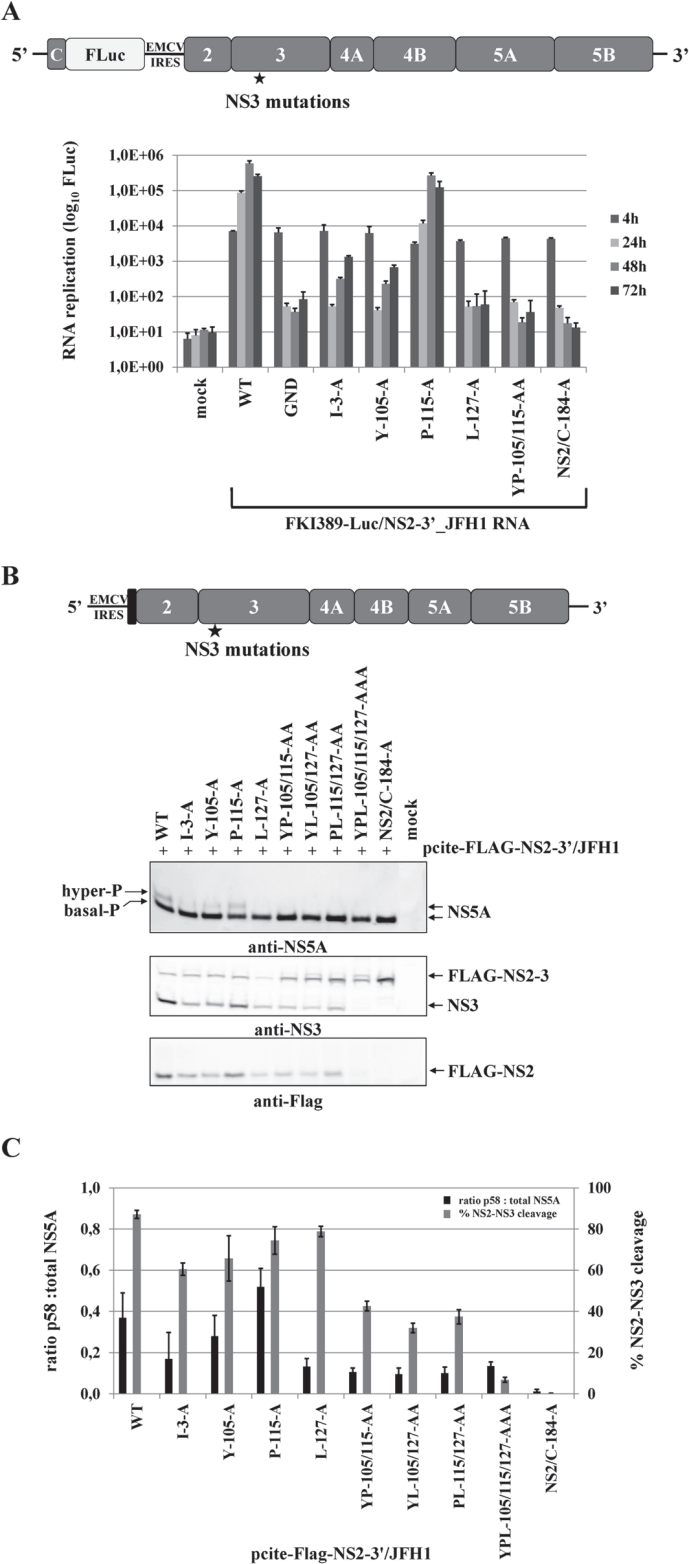
Mutational analysis of the NS3 mutations in the context of a HCV genotype 2a subgenomic NS2-5B replicon revealed NS2-NS3 cleavage is a prerequisite for NS5A hyperphosphorylation. **(A)** Top, schematic representation of the FKI389-Luc/NS2-3’_JFH1 replicon. Bottom, the NS3 mutations were introduced into pFKI389-Luc/NS2-3’_JFH1, *in vitro* transcribed RNA was electroporated into Huh7 Lunet cells, and luciferase activity was measured at 4, 24, 48 and 72 h pe. Mean values of three independent experiments are shown. Error bars indicate standard deviations. **(B)** Top, schematic of the expression construct pcite-FLAG-NS2-3’/JFH1. This plasmid encodes the T7 promotor sequence fused to the EMCV IRES followed by a FLAG epitope and NS2 to NS5B sequence of the HCV isolate 2a. Indicated NS3-cofactor mutations were engineered into pcite-NS2-3’/JFH1 and plasmids were transfected into Huh-7/T7cells infected with MVA-T7pol vaccinia virus. Bottom, effects of the NS3-cofactor mutations on NS2/3 cleavage, polyprotein processing and NS5A hyperphosphorylation in the context of NS2-5B. Cell lysates were prepared 20 h pt and analyzed by Western blotting with antibodies against FLAG-epitope, NS3 and NS5A. The positions of FLAG-NS2-3, FLAG-NS2, NS3 as well as the hyper and basally phosphorylated NS5A are indicated on the right. **(C)** Signals of Flag-NS2-3 and NS3 were quantified by ImageJ software from two Western blots to calculate the percentage of NS2-3 cleavage. Signals of NS5A p56 and p58 forms of two Western blots were quantified by ImageJ software and the ratio of hyperphosphorylation (p58) to total NS5A was calculated. WT, wild type; GND, polymerase-inactive mutant; NS2/C-184-A, NS2-protease inactive mutant; mock, cells transfected with vector control.

NS2-NS3 cleavage is essential to liberate NS3, a step that has been shown to be critical for viral RNA replication [32]. Accordingly, NS3 mutations that interfere with the NS2 protease activation in the context of the NS2-5B/JFH1 polyprotein should not only reduce the NS2-NS3 cleavage but also inhibit RNA replication similar to what has been demonstrated with NS2 mutations inactivating the NS2 protease [32]. To test this assumption, we introduced the NS3 mutations into the genotype 2a replicon pFKI389-Luc/NS2-3’ and determined the RNA replication kinetics of the mutant RNAs relative to the wild type (WT) and the non-replicative (GND) replicon RNAs. A replicon carrying a NS2-active site mutation (NS2/C-184-A) was used as a further non-replicative control which is defective in NS2-NS3 cleavage but retains its serine protease activity. The single mutations I3A, Y105A and P115A in the NS2-5B replicon RNA allowed for RNA replication to different degrees: the P115A mutant replicon RNA replicated to wild type levels whereas the mutant replicon RNAs I3A and Y105A showed a greater than 100-fold reduction in RNA replication (Fig. 5A). The L127A mutation in the NS2-5B/JFH1 replicon inhibited RNA replication to levels similar to the ones observed in the NS3-5B/JFH1 replicon. Most interestingly, the NS2-5B/JFH1 replicon with the YP105/115AA mutation that interferes with the NS2 activation by NS3 did not detectably replicate indicating that inefficient NS2-NS3 cleavage triggered by NS3 surface mutations blocks viral RNA replication (Fig. 5A).

### Efficient NS2-3 cleavage is a prerequisite for NS5A hyperphosphorylation and RNA replication in a NS2-5B replicon system

Several determinants for NS5A hyperphosphorylation have been mapped to NS3, NS4A and NS4B and expression of the NS3-5A polyprotein is required for this process. As demonstrated above, the NS3 mutation L127A strongly reduced NS5A hyperphosphorylation as well as RNA replication (Fig. 4). At the same time, L127, together with Y105 and P115, constitutes the NS3 surface patch that is required for activation of the NS2 protease in the context of uncleaved NS2-NS3 (Fig. 2). Therefore, we asked whether NS2-NS3 cleavage in a NS2-5B polyprotein is mechanistically linked to NS5A hyperphosphorylation. This hypothesis was based on the assumption that the accessibility of L127 on the NS3 surface should be compromised in the context of uncleaved NS2-NS3 with respect to its function in NS5A hyperphosphorylation. To assess the role of NS2-NS3 cleavage efficiency in regulating NS5A hyperphosphorylation experimentally, a panel of pcite-FLAG-NS2-3’/JFH1 plasmids was used for NS2-NS5B polyprotein expression in Huh-7/T7cells. For a rigorous test, a pcite-FLAG-NS2-3’/JFH1 variant carrying an NS2 protease active-site mutation (NS2/C-184-A) was used. As expected, the analysis of the NS2/C-184-A mutant showed that NS2-NS3 cleavage was abrogated without affecting polyprotein processing downstream of NS3 by the serine protease activity (Fig. 5B). In addition, the abrogation of NS2-NS3 cleavage correlated with a complete loss of NS5A hyperphosphorylation (Fig. 5B). Therefore, the NS3 release from uncleaved NS2-NS3 appears to be a prerequisite for NS5A hyperphosphorylation.

Further analyses revealed that single NS3 mutations had small but detectable effects on NS2-NS3 cleavage efficiency compared to wild type, while combinations of NS3 mutations (YP105/115AA, PL115/127AA and YL105/127AA) further decreased NS2-NS3 cleavage (Fig. 5B and 5C) and the triple mutation (YPL105/115/127AAA) almost abolished cleavage. Importantly, for double and triple mutants with decreased NS2-NS3 cleavage efficiency the analysis of NS5A revealed strongly reduced levels of hyperphosphorylated NS5A (Fig. 5B and 5C). In addition, analysis of NS5A hyperphosphorylation revealed that the small but detectable effects on NS2-NS3 cleavage efficiency for the single NS3 mutants I3A and Y105A also correlated with a reduced NS5A hyperphosphorylation compared to either wild type or P115A. In contrast, L127A decreased the production of NS5A hyperphosphorylation to levels comparable with the double or triple mutations (Fig. 5B). Thus, the effect of L127A is similar to what has been observed in the NS3-5B polyprotein. The quantification of NS2-NS3 cleavage and NS5A hyperphosphorylation indicates that an efficient NS2-NS3 cleavage increases the ratio of hyper- to basal-phosphorylation of NS5A (Fig. 5B and 5C). Together, these results strengthen our observation that NS5A hyperphosphorylation is functionally linked to NS2-NS3 cleavage in a NS2-5B polyprotein context.

### The dual function of the NS3 surface patch in stimulating NS2 activation and regulating NS5A hyperphosphorylation is conserved among HCV genotypes

**Fig 6:**
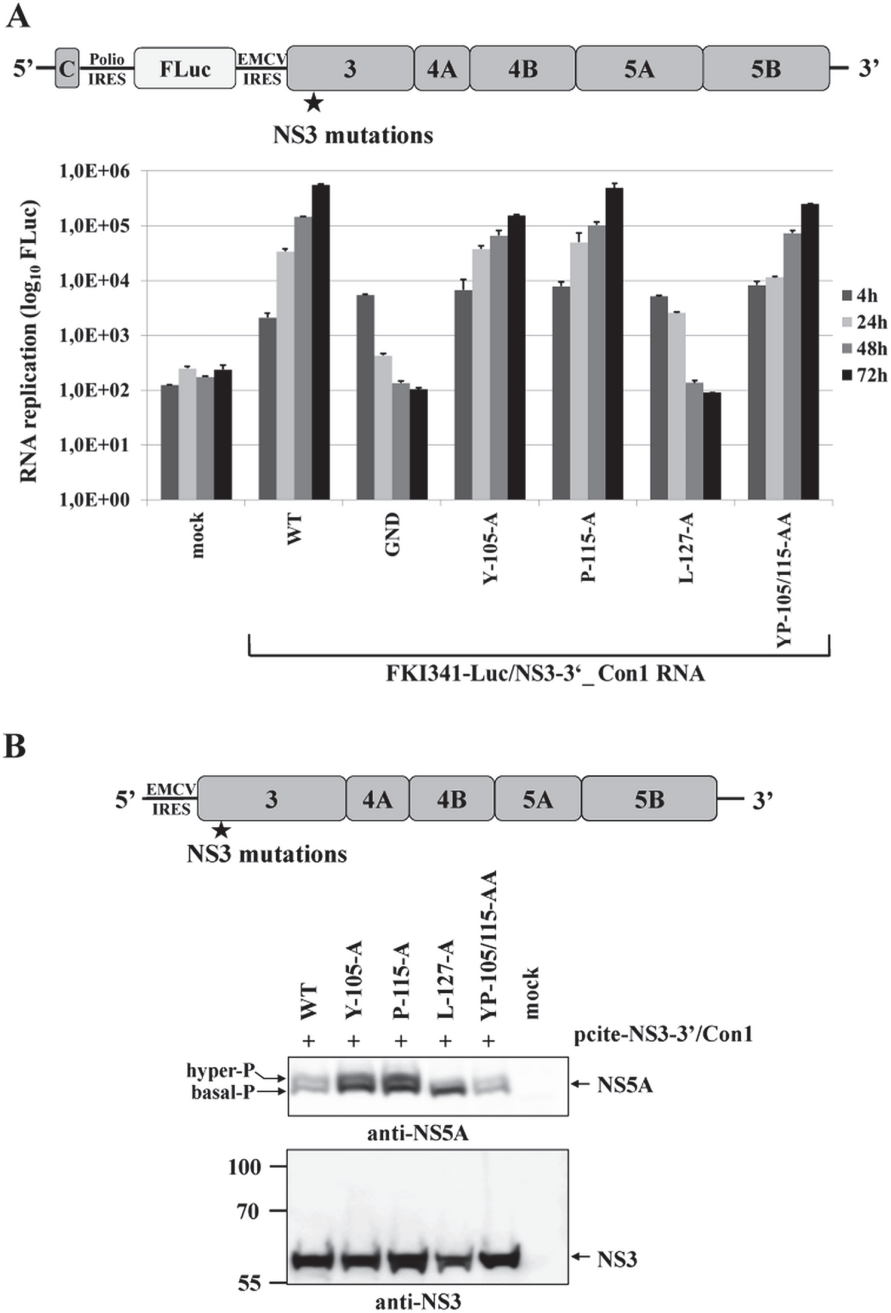
NS3-L127A inhibits RNA replication and suppresses NS5A hyperphosphorylation in the context of a HCV genotype 1b NS3-5B replicon. **(A)** Top, schematic of the HCV genotype 1b NS3-5B replicon. Bottom, the NS3 mutations were engineered into pFKI341-Luc/NS3-3’_Con1 (WT), transcribed RNA was electroporated into Huh7 Lunet cells. Luciferase activity was measured at 4, 24, 48 and 72 h pe. Mean values of three independent experiments are shown, error bars indicate standard deviations. **(B)** Top, schematic of pcite-NS3-3’/Con1. This plasmid encodes the NS3 to NS5B sequence of the HCV isolate 1b (Con1). NS3 mutations were introduced into pcite-NS3-3’/Con1 and plasmids were transfected into Huh-7/T7cells infected with MVA-T7pol vaccinia virus. Bottom, effects of the NS3 mutations on NS3-5B polyprotein processing and NS5A hyperphosphorylation. Cell lysates were prepared 20 h pt and analyzed by Western blotting with anti NS3 and NS5A antibodies. The position of hyper and basally phosphorylated NS5A is indicated on the left. WT, wild type; GND, polymerase-inactive mutant; mock, cells transfected with vector control.

Recent work characterizing NS5A hyperphosphorylation in replicons of HCV genotype 1b (Con1) and genotype 2a (JFH1) isolates revealed significant differences concerning the degree of correlation between NS5A hyperphosphorylation and viral RNA replication [33–36]. Therefore, we determined if L127 is also critical for NS5A hyperphosphorylation in the context of the NS3-5B/Con1 replicase. Accordingly, the NS3 mutations were introduced into a NS3-5B/Con1 luciferase reporter replicon and analyzed for their effect on RNA replication (Fig. 6A). NS3-5B/Con1 polyprotein processing as well as the NS5A phospho-form distribution were determined by Western blotting using Huh-7/T7 cells and the MVA-T7pol vaccinia virus system (Fig. 6B). In agreement with the results obtained for the NS3-5B/JFH1 replicon, the NS3-5B/Con1 replicons encoding for the NS3 mutants Y105A, P115A and YP105/115AA showed robust RNA replication, comparable to wild type, while a replicon encoding the NS3 L127A exchange was replication-deficient (Fig. 6A). Transient protein expression in cells transfected with the corresponding set of mutant pcite-NS3-5B constructs followed by immunoblotting assays did not reveal a substantial inhibition of the NS3-5B/Con1 polyprotein processing indicating that no apparent defect in the NS3-NS4A serine protease was induced by the NS3 mutations (Fig. 6B). In agreement with our results for JFH1, only the substitution L127A significantly reduced the ratio of hyper- to basal phosphorylated NS5A relative to wild type in the context of the NS3-5B/Con1 polyprotein (Fig. 6B).

**Fig 7:**
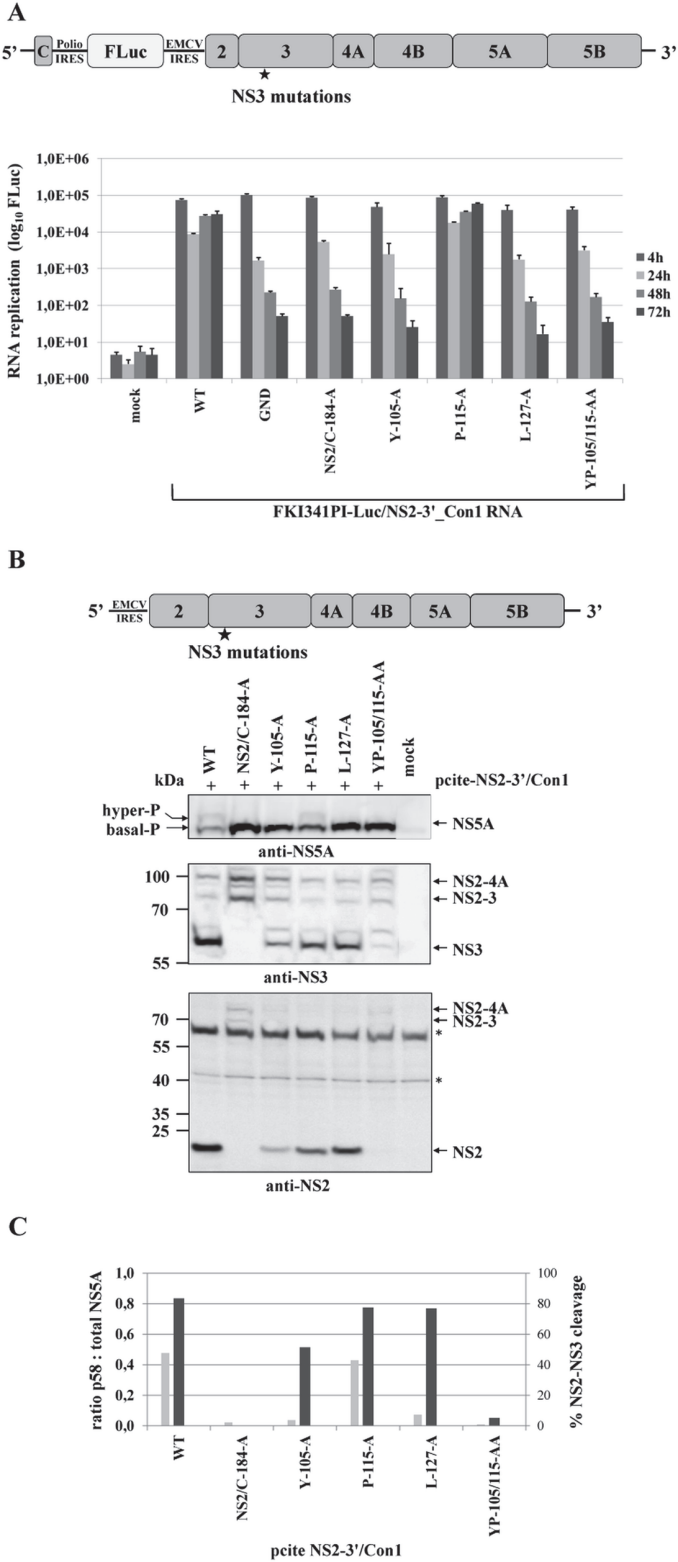
Mutational analysis of NS3 surface residues in a HCV genotype 1b NS2-5B replicon confirm the critical role of efficient NS2-NS3 cleavage for NS5A hyperphosphorylation. **(A)** Top, schematic of the HCV genotype 1b NS2-5B replicon. Bottom, the NS3 mutations were built into pFKI341-Luc/NS2-3’_Con1 and transcribed RNA was electroporated into Huh7 Lunet cells. Luciferase activity was measured at 4, 24, 48 and 72 h pe. Mean values of three independent experiments are shown. Error bars indicate standard deviations. **(B)** Top, schematic of pcite-NS2-3’/Con1. This plasmid encodes the NS2 to NS5B sequence of the HCV isolate 1b as indicated. NS3 mutations were introduced into pcite-NS2-3’/Con1 and plasmids were transfected into Huh-7/T7cells infected with MVA-T7pol vaccinia virus. Bottom, effects of the NS3 mutations on NS2-5B polyprotein processing and NS5A hyperphosphorylation. Cell lysates were prepared 20 h pt and analyzed by Western blotting with antibodies directed against NS2, NS3 and NS5A. The position of hyper and basally phosphorylated NS5A is indicated. **(C)** Signals of NS2-3 and NS3 were quantified by ImageJ software from two Western blots to calculate the percentage of NS2-3 cleavage. Signals of NS5A p56 and p58 forms of two Western blots were quantified by ImageJ software and the ratio of hyperphosphorylation (p58) to total NS5A was calculated. WT, wild type; GND, polymerase-inactive mutant; NS2/C-184-A, NS2-protease inactive mutant; mock, lysates from cells transfected with vector control.

Next, we also tested in the Con1 replicon system if a correlation between the NS2-NS3 cleavage efficiency and NS5A hyperphosphorylation exists. To this end we first determined the replication capacities of mutant NS2-5B/Con1 replicons in a transient replication assay. Among the mutant NS2-5B/Con1 replicons that were examined only the one encoding the NS3 P115A exchange replicated to wild type level (Fig. 7A). All other NS2-5B/Con1 replicons examined did not replicate to detectable levels. The observation that the NS2-5B/Con1 replicons with the NS2/C184A, the YP105/115AA and the L127A mutations showed no replication corroborated our earlier observations in the NS2-5B/JFH1 replicon system. The fact that the NS2-5B/Con1 Y105A replicon derivative did also fail to replicate was somewhat unexpected and differs from the Y105A NS2-5B/JFH1 mutant replicon that still replicates at low level compared to WT (Fig. 5A). Processing of the respective Y105A NS2-5B/Con1 polyprotein upon expression by the MVA-T7 pol vaccinia virus system revealed that the NS2-NS3 cleavage was already strongly reduced compared to either wild type or the NS3 P115A mutant NS2-5B (Fig. 7B and 7C). Moreover, in cells expressing the NS2-5B/Con1 Y105A polyprotein, NS5A hyperphosphorylation was almost undetectable in our Western blot system when compared to the wild type or NS2-5B/Con1 (P115A) polyprotein (Fig. 7B and 7C). Together, these findings again emphasize the functional link between NS2-NS3 cleavage efficiency and NS5A hyperphosphorylation.

Collectively, these results point to a conserved and, so far, unappreciated role of efficient NS2-NS3 cleavage as a prerequisite for the NS5A hyperphosphorylation during the biogenesis of the HCV replication complex in different HCV genotypes in a process that is critical for viral genome replication.

### The hydrophobic NS3 surface residues required for NS2 protease stimulation are not critical for NS2/NS3 interactions and virus assembly in the context of a bicistronic genome

NS2 is required for virion assembly and this function has been assigned to both the N-terminal membrane association- and the C-terminal protease domain [19,21,22,37,38]. Accordingly, the NS2 protease domain not only catalyzes the NS2-NS3 cleavage but also provides determinants for virion morphogenesis. This observation together with the finding that mature NS2 is interacting with NS3 in a way that has been implicated to coordinate virion assembly [24], prompted us to test whether mutations in the hydrophobic NS3 surface patch have effects on infectious virus production. To this end we introduced the NS3 mutations Y105A, P115A, L127A and YP105/115AA into the JFH1ad-R2a_NS2EI3 genome (S4 and S5 Figs.). In this bicistronic context, virus assembly can be analyzed independently from NS2-NS3 cleavage and thus uncouples NS2-NS3 processing from replication and virus assembly. Both single mutations replicated to wild type level, whereas the double mutation (YP105/115AA) exhibited slightly reduced replication (S4B Fig.). The L127A mutation did not replicate in this bicistronic context (S5 Fig.) confirming our earlier observation that this mutation inhibited RNA replication (Figs. 4A and 5A). The replication-competent NS3 mutants (Y105A, P115A and YP105/115AA) have no detectable effect on NS5A hyperphosphorylation in the context of the bicistronic JFH1ad-R2a_NS2EI3 genome (S6 Fig.) as expected from our transient expression experiments with the NS3-5B polyprotein (Fig. 4B). Infectious virus production was similar to wild type for both single mutants, while the double mutant showed a reduction of infectious titers of about 10-fold (S4C and S4D Fig.). When we calculated the infectivity release efficiency as a ratio of infectivity release and replication the double mutation exhibited a 5-fold reduction compared to wild type, whereas the single mutations showed efficiencies of about 60% of wild type level (S4E Fig.).

The double mutant YP105/115AA strongly reduces NS2 protease stimulation by NS3 (Fig. 5B) most likely by disturbing surface interactions between NS2 and NS3 in the NS2-NS3 precursor protein. The same mutations mildly reduce the infectivity release efficiency to 20% of wild type in a bicistronic background (S4E Fig.). We also examined whether the NS3 mutations influence binding between NS2 and NS3, which could, in case of the double mutant YP105/115AA, explain the observed moderate reduction in virus production. To this end, Huh7.5 cells were transfected with wild type JFH1ad_HAF-NS2EI3 (carrying a HA-Flag epitope sequence, HAF, in NS2; S7A Fig.) or mutant RNAs encoding individual NS3 substitutions. After 72 hours cells were harvested, lysed and lysates were used for immunoprecipitation. Western blot analysis of immunoprecipitated HAF-NS2 protein as well as the co-immunoprecipitated NS3 indicated that the mutations in NS3 did not detectably influence NS2:NS3 interaction (S7B Fig.). Together, these results showed that virus mutants with substitutions in the hydrophobic NS3 surface patch are still capable of virus production albeit at slightly (Y105A and P115A) or moderately (YP105/115AA) reduced levels compared to wild type. This indicates that the hydrophobic NS3 surface residues required for NS2 protease stimulation are not critical for virus assembly in the context of a bicistronic genome.

## Discussion

Polyprotein processing is a key step in the HCV life cycle and the NS2-NS3 autocleavage has been demonstrated to be essential for RNA replication of full-length virus and subgenomic NS2-5B replicons [18,32]. NS2 in its cleaved form is also required for virion morphogenesis [19,22,37–39]. Previous studies have shown that the NS2 protease activity critically depends on the presence of the NS3 cofactor [17] and that the formation of an NS2 dimer is required for NS2-NS3 cleavage due to the unique composite nature of the NS2 protease active site [12,16,20]. Since the structure of the NS2-NS3 protein in its pre-cleavage conformation has not been determined, the molecular mechanism of the NS2-activation and its regulation remained enigmatic.

Our finding that a conserved hydrophobic NS3 surface patch composed of Y105, P115 and L127 is essentially involved in NS2 protease stimulation reveals that NS2 protease stimulation is mainly directed by specific hydrophobic NS3 surface residues (Fig. 2). This observation is supported by our mutational characterization of this surface area. While the introduction of single charged amino acids in this hydrophobic core strongly reduces (P115R) or abolishes (Y105R, L127R) NS2 protease activation, more conservative phenylalanine exchanges of Y105, P115 and L127 were tolerated with regard to NS2-NS3 cleavage (Fig. 2B). Accordingly, the hydrophobic character of this surface patch is pivotal for the NS3 cofactor function in NS2 protease activation. The finding that only combinations of these amino acids inhibit NS2-NS3 cleavage suggests that multiple hydrophobic NS3 residues are required to create a continuous hydrophobic NS3 surface area that interacts with NS2 to stimulate NS2 protease activity (Fig. 2A). This NS2/NS3 interaction in uncleaved NS2-NS3 likely promotes the efficient formation of a functional conformation required for productive NS2-NS3 autoprocessing. Interestingly, the NS3 surface area implicated in the formation of a proteolytically active conformation of the NS2-NS3 complex is situated in the previously defined NS3 Zn^2+^-binding domain [17]. This domain was shown to represent the functional NS2-protease activating domain and can also activate NS2-NS3 cleavage *in trans* [17]. Our results indicate that intrinsic structural preferences in the NS2-NS3 precursor have important regulatory roles in the NS2-NS3 autocleavage reaction. Mechanistically, it has been proposed that the intramolecular NS2-NS3 cleavage is performed by active NS2-NS3 dimers in the course of co- and posttranslational polyprotein processing [40]. Although critical residues for NS2 dimerization are not known [16], the detection of NS2 interactions in mammalian cells by co-immunoprecipitation in the absence of NS3 suggests that NS3 is not essential for NS2 dimer formation [40]. In theory, the formation of a proteolytically active NS2-NS3 precleavage complex could be promoted by either intermolecular or intramolecular interactions between NS2 and NS3 (i.e., between the NS2 moiety of one molecule of NS2-NS3 and the NS3 moiety of a second NS2-NS3 molecule or intramolecular of NS2-NS3 molecules which form dimers). Future experiments will aim at the identification of corresponding interaction sites on NS2 and to investigate if the stimulation of NS2 by NS3 in uncleaved NS2-NS3 is based on intra- or intermolecular protein interactions. Such regulation of polyprotein processing is also seen in other polyprotein-encoding viruses. For instance, the regulation of P23 polyprotein processing in alphaviruses is achieved by extensive intramolecular contacts between nsP2 and nsP3 which may function in positioning and recognition of the P2/3 cleavage site [41,42]. In contrast, poliovirus 3CD has no intramolecular contacts and cleavage of the solvent exposed cleavage site is regulated by intermolecular contacts between 3CD molecules [43].

A major finding of this study is that the hydrophobic NS3 surface involved in activating the NS2 protease has a second important function in the regulation of viral RNA replication. The L127A mutation in NS3 massively reduced NS5A hyperphosphorylation and this defect strongly correlated with an inhibition of viral RNA replication of NS3-5B replicons of different HCV genotypes without affecting the NS3-4A serine protease activity (Figs. 4 and 6). Determinants for NS5A hyperphosphorylation have been mapped to NS3, NS4A and NS4B and expression of the NS3-5A polyprotein is required for this process [44–47]. These findings suggest that the molecular basis for the multifactorial nature of NS5A hyperphosphorylation is its dependency on the assembly of the viral replicase.

These results point to a surprising functional overlap of NS3 surface determinants that are involved in NS2 protease activation and NS5A hyperphosphorylation, a process depending on viral replicase assembly, with L127 being a structural constituent of both NS3 functions. The dual role of L127 in promoting NS2-NS3 cleavage and regulating NS5A hyperphosphorylation prompted us to test whether NS2-NS3 cleavage is a prerequisite for NS5A hyperphosphorylation. The detailed mechanism underlying the NS5A hyperphosphorylation is still poorly understood [34,35,48,49]. It was hypothesized that the generation of authentic NS2 by NS2-NS3 cleavage is important for NS5A hyperphosphorylation [50]. However, hyperphosphorylated NS5A was also detected in the absence of NS2 suggesting that the authentic N-terminus of NS3, generated by NS2-NS3 cleavage, represents the critical determinant for NS5A hyperphosphorylation [44,51]. We could demonstrate that mutations either inactivating the NS2 protease (NS2 C184A) or blocking the NS2 protease cofactor function of NS3 (YP105/115AA) were blocking or strongly reducing NS5A hyperphosphorylation in NS2-5B polyproteins of two different HCV genotypes. Reductions in the NS2-NS3 cleavage efficiency correlated not only with a decrease in NS5A hyperphosphorylation but also with a block in viral RNA replication of the respective NS2-5B replicons (Figs. 5 and 7). This correlation became apparent by using the NS2-5B replicons of genotype 1b and 2a. When we compared the impact of the NS3 mutations on RNA replication in these systems, we observed that the Y105A mutation inhibited NS2-5B/Con1 replication but was still allowing for RNA replication in the NS2-5B JFH1 background (compare Figs. 5A and 7A). This difference is most likely due to the significantly lower replication capacity of the genotype 1b replicon RNA and is also reflected by the detectable NS5A hyperphosphorylation only in the case of the NS2-5B/JFH1 (Figs. 5B and 7B). These results are in line with the recent observation that, although NS2-NS3 cleavage is not limiting Con1 replication, the formation of the membranous HCV replication complexes (RC) might be less efficient in Con1 compared to the biogenesis of JFH1 RCs [52].

Based on our data, we propose the following order of events: NS2 in uncleaved NS2-NS3 interacts with the hydrophobic NS3 surface area that includes L127 resulting in NS2 protease stimulation. As a consequence, in uncleaved NS2-NS3 the NS3 surface surrounding L127 is most likely not accessible for protein-protein interactions since this region is engaged in interactions with the NS2 protease domain. Upon NS2 release the NS3 surface area around L127 becomes available for novel protein-protein interactions that finally allow NS5A hyperphosphorylation to occur. We propose that this process is functionally linked to the assembly of the viral replicase complex and likely involves interactions of NS3 with NS4A [32,47]. Moreover, after NS2 release NS3 can undergo a structural change to adopt a conformation required for its function within the viral replicase. These changes are suggested to promote inter-domain co-operations between NS3 helicase and serine protease domain [53] and lead to efficient NS4A binding [32]. Interestingly, such a scenario is supported by the observation that uncleaved NS2-NS3 exhibits a lower affinity to NS4A when compared to NS3 [32]. In this context it is remarkable that mutations in the NS4A acidic domain could be rescued by second site mutations in the NS3 protease domain [47]. One of these residues is in close proximity to L127 on the NS3 surface supporting our finding that this region is critical for NS5A hyperphosphorylation and likely for the assembly of the viral replication complex. Together, our data indicate that NS2-NS3 cleavage is mechanistically linked to NS5A hyperphosphorylation as well as the assembly of the viral replicase. These intriguing observations reveal an unexpected function for the NS2-NS3 cleavage and might explain why an efficient liberation of functional NS3 is a prerequisite for viral genome replication.

The identification of a hydrophobic surface segment on NS3 as an essential module of the NS2 protease cofactor in NS3 which also plays a critical role in replicase assembly is an important step towards a better understanding of the sequential processes involved in the functional assembly of the HCV RNA replication complex at the molecular level which are most certainly accompanied by conformational transitions within the different macromolecular complexes.

## Methods

### Cell culture

Huh7 Lunet [44] and Huh7/T7 [54] cells were maintained in Dulbecco's modified minimal essential medium supplemented with 10% FCS, 100 U penicillin/100 μg/ml streptomycin, and 2 mM L-glutamine. Huh-7/T7cells were cultured in the presence of 400 μg/ml G418.

### Plasmids and mutagenesis

Genomes Con1 [44,55], BK [56], JFH1 [57] have been described. All mutations were introduced via QuikChange (QC) mutagenesis. Details concerning the generation of constructs and their properties can be found in the supplementary material.

### In vitro transcription and electroporation of HCV RNAs

The experimental procedures used to generate *in vitro* transcripts from cloned HCV sequences and transfection of Huh-7 cells by electroporation have been described [37]. After electroporation, cells were immediately transferred to complete DMEM and seeded as required for the assay.

### Luciferase assay

At each time point (4, 24, 48, and 72 h), cells were washed with PBS, scraped into 1 ml of PBS and collected by centrifugation. The cells were lysed in 40 μl of lysis buffer (PJK-GmbH). 20 μl of the lysate was analyzed using the Beetle Juice luciferase assay system (PJK-GmbH) and measured in a luminometer (Junior LB9509, Berthold).

### Vaccinia virus infection, DNA transfection and transient protein expression

The applied procedures have been described [58]. HCV nonstructural proteins were expressed from pcite plasmids. Briefly, 2 x 10^6^ Huh-7/T7 cells were infected with MVA-T7pol vaccinia virus [59] and subsequently transfected with 4 or 8 μg of plasmid DNA by using Superfect reagent (QIAGEN).

### SDS-PAGE and western blotting

Proteins were separated in polyacrylamide-Tricine gels. After SDS-PAGE, proteins were transferred onto a nitrocellulose membrane (Pall, USA). The membrane was blocked with 5% (w/v) dried skim milk in phosphate-buffered saline with 0.05% (v/v) Tween 20 (Invitrogen). For antigen detection, anti-NS5A 9E10 [60], mouse monoclonal antibody against NS3 of the JFH-1 isolate (4D11) (generated in a cooperation between Harish Ramanathan, Michael Engle, Michael S. Diamond and Brett D. Lindenbach) or anti-NS3 (2E3) [61], anti-NS2 (YAL-4-70-8, Cell Essentials), anti-NS4B [62], anti-FLAG (Sigma), anti-V5 (Invitrogen), anti-HA (HA.11 clone 16B12, Covance) and anti-GST (GE Healthcare), antibodies were used in 2% (w/v) dried skim milk in phosphate-buffered saline with 0.05% (v/v) Tween 20. For primary antibody detection, horseradish peroxidase-conjugated species-specific secondary antibodies (Dianova) were used at a 1:3000 dilution and Western Lightning Chemiluminescence Reagent Plus (Perkin Elmer) was applied prior to imaging using a LAS 4000 imaging system (Biorad, Munich). Quantifications of Western blots were carried out using ImageJ 1.47t software (NIH, Bethesda).

### Metabolic labeling of proteins

HCV nonstructural proteins were expressed from pcite plasmids. For transient protein expression, 2 x 10^6^ Huh-7/T7 cells were infected with MVA-T7pol vaccinia virus [59] and subsequently transfected with 4 μg of plasmid DNA by using Superfect reagent (QIAGEN). Cells were kept in DMEM culturing medium for 2 h. Medium was changed to DMEM (lacking L-Methionine, L-Cysteine and L-Glutamine) supplemented with 1% Glutamax (Gibco). After 30 min the medium was changed again for DMEM (lacking L-Methionine, L-Cysteine and L-Glutamine) supplemented with 1% Glutamax (Gibco), containing 70 μCi (1Ci = 37GBq) [^35^S]-labeled methionine/ cysteine (Hartmann Analytics).

### RIP

After 6 h cells were lysed in 250 μl RIPA(G) [150 mM NaCl, 1% (vol/vol) Nonidet NP40, 0.5% (wt/vol) deoxycholate, 0.1% (wt/vol)SDS, 50 mM Tris (pH 8)], containing 1 mM Pefabloc^SC^ (Roth). The following steps were performed at 4°C, mixing was ensured by placing samples on a spinning wheel. Lysates were incubated for 30 min and then centrifuged for 30 min at 16.000 g. The supernatants were incubated with anti-GST antibody (GE Healthcare) 1:400 in RIPA(G) for 1 h, then 50 μl of a 20% (vol/vol) Protein-A-Sepharose suspension were added and incubated for another hour. The Protein-A-Sepharose was pelleted at 16.000 g and washed 3 x with RIPA(G). Proteins were denatured in sample buffer containing 5% β-Mercaptoethanol at 95°C for 10 min and then separated in polyacrylamide-tricine gels with 8% polyacrylamide. SDS-Gels were fixed in a solution containing 40% methanol and 10% acetic acid, dried and exposed to Imaging screens (Fuji) for 1–3 days. Readouts were performed using Phosphorimager (Fuji BAS). Quantifications were carried out with AIDA Image Analyzer (Version 3.52) software using a background substraction method.

## Supporting Information

S1 Fig Effect of selected NS3 double mutations on NS3-4A serine protease activity. (A) Scheme of the HCV NS3-4A serine protease assay. NS3-4A-mediated cleavage of co-expressed FLAG-MBP-NS4B-NS5A-trx-HA/BK serine protease substrate results in the generation of FLAG-MBP-NS4B and NS5A-trx-HA cleavage products. (B) NS3-4A serine protease assay. Plasmids with the indicated amino acid mutations were expressed and NS4B-NS5A cleavage was detected by Western blotting. WT refers to wild type NS3(1–172), pcite-V5-NS4A/BK refers to a plasmid expressing the V5-tagged NS4A cofactor, pcite-FLAG-MBP-NS4B-NS5A-trx-HA/BK indicates a plasmid expressing the serine protease cleavage substrate FLAG-MBP-NS4B-NS5A-trx-HA. Mock indicates cells transfected with a vector control. The positions of the NS3(1–172)GST, V5-NS4A, FLAG-MBP-NS4B-NS5A-trx-HA, FLAG-MBP-NS4B and NS5A-trx-HA proteins are indicated by arrows. (C) Signals of FLAG-MBP-NS4B-NS5A-trx-HA and NS5A-trx-HA were quantified by ImageJ software from two Western blots to calculate the percentage of FLAG-MBP-NS4B-NS5A-trx-HA cleavage as an indication of the NS3 serine protease activity.
(TIF)

S2 Fig A conserved hydrophobic NS3 surface patch consisting of residues Y105, P115 and L127 is important for NS2-activation by NS3. Location of NS3 residues identified in the di-alanine scan to inhibit the NS2-NS3 cleavage in the NS3 structure of the genotype 1b. The di-alanine mutations that specifically inhibit NS2 activation by NS3 (IT3/4, LY104/105 and IP114/115) are shown in green stick representation. The position of LL126/127 is marked in light green stick representation to indicate the reduced NS3 serine activity of this mutant in the NS4B-NS5A *trans* cleavage assay. The NS3 residues LV106/107, PL142/143, GI152/153 and DF168/169 identified in the di-alanine scan to inhibit the NS2 activation by NS3 as well as the NS3 serine protease activity in a NS4B-NS5A *trans* cleavage assay are shown in red stick representation. The overall NS3 structure is shown in grey surface representation. Carbon and backbone ribbon are colored in yellow for the protease domain and blue for the helicase domain, respectively. An enlargement of the NS3 is shown on the right. The active site of the NS3 serine protease is indicated. The figure was generated using Pymol version 1.10 and the coordinates of PDB code 1CU1 [31].
(TIF)

S3 Fig The hydrophobic character of the NS3 surface area is important for the NS2 protease stimulation by NS3. The cleavage efficiencies of the indicated BK NS2-NS3 polyprotein fragments carrying either charged (arginine shown in panel A) or hydrophobic (phenylalanine shown in panel B) NS3 amino acid substitutions were determined by Western blot analysis. Western blot signals of Flag-NS2-3(1–172)GST and Flag-NS2 of two independent Western blots were quantified by ImageJ software and the percentage of NS2-NS3 cleavage was calculated.
(TIF)

S4 Fig Impact of amino acid substitutions in NS3 protein on the HCV replication and infectivity release. (A) Schematic diagram of the full length JFH1ad-R2a_NS2EI3 genome used for functional characterization. The HCV proteins and NTRs are represented as white boxes, renilla luciferase (RenLuc) used for indirect quantitative analysis of HCV genome is shown in dark gray. FMDV 2A peptide sequence in light gray facilitates release of authentic core protein. The EMCV IRES sequence was inserted between NS2 and NS3 sequences to separate protease activity from replication and is shown as a black box. Cell culture titer enhancing mutations in NS5A and NS5B (V2153A, V2440L and V2941M) are represented as white stars. The positions of NS3 aa substitutions Y105A and P115A are indicated as a black star. (B) Huh7.5 cells were transfected with viral RNA specified at the bottom of the graph and kinetics of HCV genome replication were quantified 24, 48 and 72 hours post transfection by luciferase assay. (C) Supernatants of transfected cells containing released infectious particles were harvested 24, 48 and 72 hours post transfection and used for infection of naïve Huh7.5 cells. After three days, infected cells were lysed and intracellular luciferase activity was measured. (D) Huh7.5 cells were transfected with RNAs specified at the bottom of the graph and release of infectious particles into culture supernatants were quantified 24, 48 and 72 hours post transfection by TCID50 assay. In panels (B), (C) and (D) representative results of two independent experiments performed in duplicates with standard deviations are shown. Dotted lines represent background values of the assays. (E) Efficiency of infectivity release was estimate as a ratio of infectivity release (luciferase value of graph C) and replication (luciferase value of graph B).
(TIF)

S5 Fig Impact of L127A amino acid substitution in NS3 protein on the JFH1ad-R2a_NS2EI3 replication and infectivity release. (A) Huh7.5 cells were transfected with viral RNA specified at the bottom of the graph or mock transfected and kinetics of HCV genome replication were quantified 4, 24, 48 and 72 hours post transfection by luciferase assay. (B) Supernatants of transfected cells containing released infectious particles were harvested 24, 48 and 72 hours post transfection and used for infection of naïve Huh7.5 cells. After three days, infected cells were lysed and intracellular luciferase activity was measured. (C) The kinetics of replication (data from panel A) was determined by normalizing the relative light units at the different time points to the mock value and the respective 4-h value. In panels (A) and (B) representative results of two independent experiments performed in duplicates with standard deviations are shown. Dotted lines represent background values of the assays measured with blank buffer.
(TIF)

S6 Fig Substitution of Y105A or P115A amino acid in NS3 protein does not have significant impact on the NS5A protein phosphorylation in the context of infectious full-length virus JFH1ad_HAF-NS2EI3. (A) Huh7.5 cells were transfected with viral RNA specified at the bottom or mock transfected, the cell lysates were harvested 72 hours post transfection and analyzed by Western Blot assay. (B) Western blot signals of NS5A p56 and p58 forms of six independent Western blots from two biological repetitions were quantified by Quantity One software and the ratio of p58 and p56 was calculated.
(TIF)

S7 Fig Amino acid substitutions in the NS3 protein do not significantly affect interaction with NS2 protein in the full length bicistronic viral context. (A) A schematic diagram of the full length JFH1ad_HAF-NS2EI3 genome used for NS2/NS3 proteins co-immunoprecipitation. The HCV proteins and NTRs are represented as white boxes. EMCV IRES sequence inserted between NS2 and NS3 sequences and HA and Flag tag sequences are shown as black boxes. Cell culture titer enhancing mutations in NS5A and NS5B (V2153A, V2440L and V2941M) are represented as white stars. Position of NS3 aa substitutions Y105A and P115A is shown as a black star. (B) Huh7.5 cells were transfected with wt or genome containing an NS3 aa substitution and harvested after 72 hours and lysed. The HCV construct without HA-Flag tag sequence (JFH1ad_NS2EI3) and mock-transfected cells were used as technical negative controls. Protein samples were used for HA specific immunoprecipitation. Pull down efficiency of HAF-NS2 protein as well as co-immunoprecipitated NS3 protein were analyzed by Western Blot assay. Input lysate and sample containing immunoprecipitated proteins were loaded on the gel in the ration 1:10.
(TIF)

S1 Text Supporting materials and methods. Supporting reference list.
(DOC)
