# Correction: A Conserved NS3 Surface Patch Orchestrates NS2 Protease Stimulation, NS5A Hyperphosphorylation and HCV Genome Replication

**DOI:** 10.1371/journal.ppat.1005394

**Published:** 2016-01-08

**Authors:** Olaf Isken, Ulrike Langerwisch, Vlastimil Jirasko, Dirk Rehders, Lars Redecke, Harish Ramanathan, Brett D. Lindenbach, Ralf Bartenschlager, Norbert Tautz

The following information is missing from the Funding section: HR and BDL were funded by United States’ Public Health Service Grant R01 AI089826.
