# Supplementary figures and images for: A Conserved NS3 Surface Patch Orchestrates NS2 Protease Stimulation, NS5A Hyperphosphorylation and HCV Genome Replication
Source: PLoS Pathog. 2015 Mar 16;11(3):e1004736. doi: 10.1371/journal.ppat.1004736 (PMC4361677; doi:10.1371/journal.ppat.1004736)

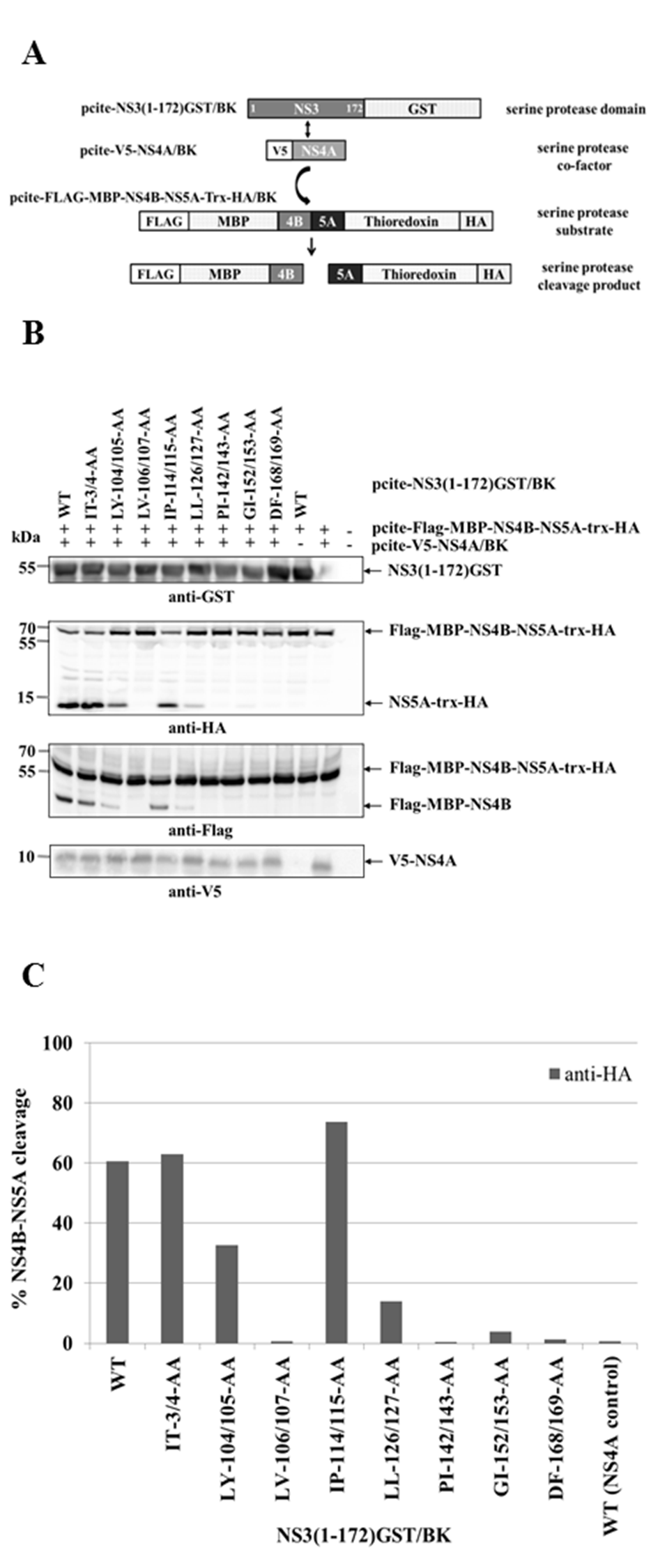

Supplement: S1 Fig — (A) Scheme of the HCV NS3-4A serine protease assay. NS3-4A-mediated cleavage of co-expressed FLAG-MBP-NS4B-NS5A-trx-HA/BK serine protease substrate results in the generation of FLAG-MBP-NS4B and NS5A-trx-HA cleavage products. (B) NS3-4A serine protease assay. Plasmids with the indicated amino acid mutations were expressed and NS4B-NS5A cleavage was detected by Western blotting. WT refers to wild type NS3(1–172), pcite-V5-NS4A/BK refers to a plasmid expressing the V5-tagged NS4A cofactor, pcite-FLAG-MBP-NS4B-NS5A-trx-HA/BK indicates a plasmid expressing the serine protease cleavage substrate FLAG-MBP-NS4B-NS5A-trx-HA. Mock indicates cells transfected with a vector control. The positions of the NS3(1–172)GST, V5-NS4A, FLAG-MBP-NS4B-NS5A-trx-HA, FLAG-MBP-NS4B and NS5A-trx-HA proteins are indicated by arrows. (C) Signals of FLAG-MBP-NS4B-NS5A-trx-HA and NS5A-trx-HA were quantified by ImageJ software from two Western blots to calculate the percentage of FLAG-MBP-NS4B-NS5A-trx-HA cleavage as an indication of the NS3 serine protease activity. (TIF) [file ppat.1004736.s001.tif]

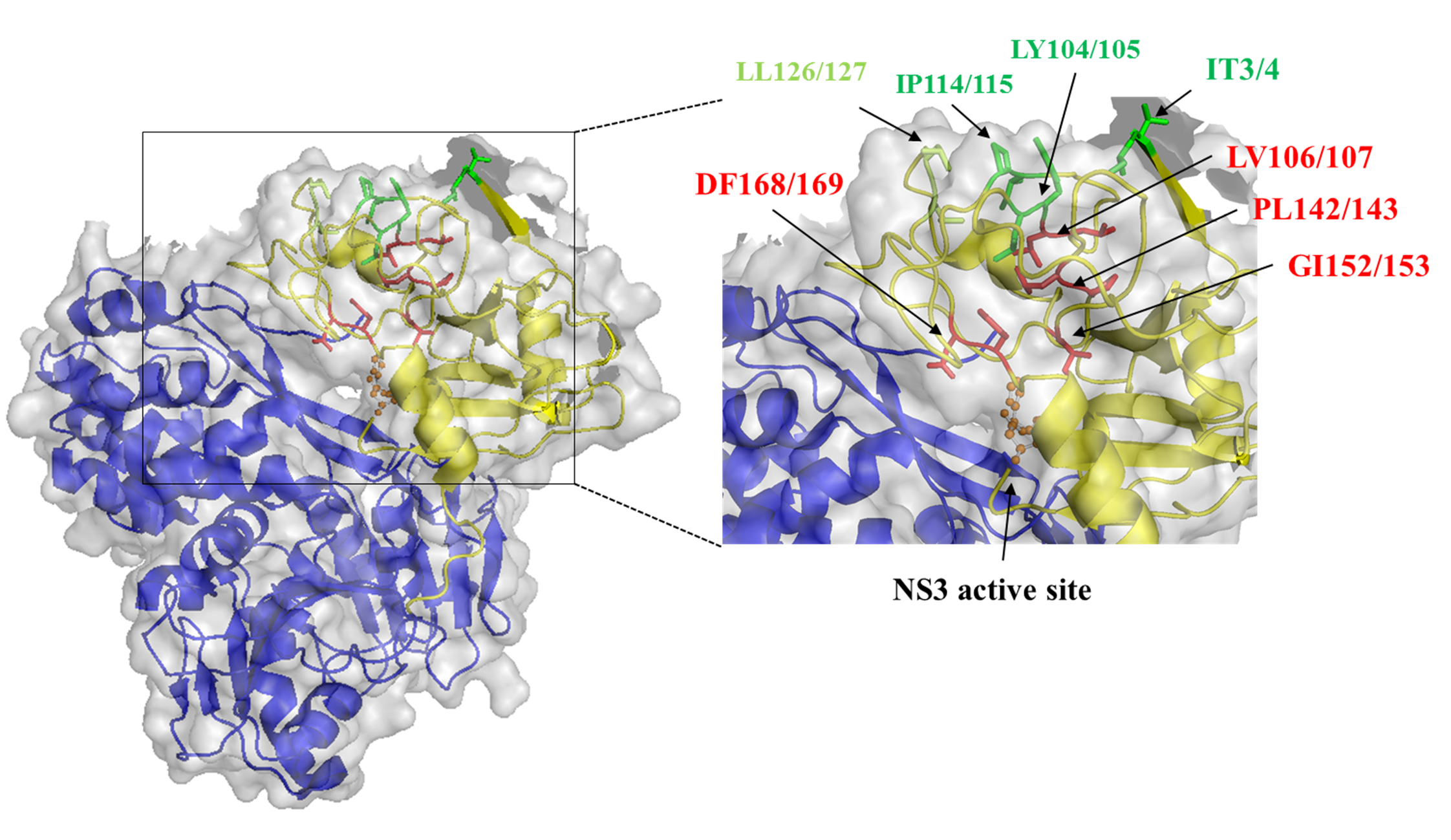

Supplement: S2 Fig — Location of NS3 residues identified in the di-alanine scan to inhibit the NS2-NS3 cleavage in the NS3 structure of the genotype 1b. The di-alanine mutations that specifically inhibit NS2 activation by NS3 (IT3/4, LY104/105 and IP114/115) are shown in green stick representation. The position of LL126/127 is marked in light green stick representation to indicate the reduced NS3 serine activity of this mutant in the NS4B-NS5A trans cleavage assay. The NS3 residues LV106/107, PL142/143, GI152/153 and DF168/169 identified in the di-alanine scan to inhibit the NS2 activation by NS3 as well as the NS3 serine protease activity in a NS4B-NS5A trans cleavage assay are shown in red stick representation. The overall NS3 structure is shown in grey surface representation. Carbon and backbone ribbon are colored in yellow for the protease domain and blue for the helicase domain, respectively. An enlargement of the NS3 is shown on the right. The active site of the NS3 serine protease is indicated. The figure was generated using Pymol version 1.10 and the coordinates of PDB code 1CU1 [31]. (TIF) [file ppat.1004736.s002.tif]

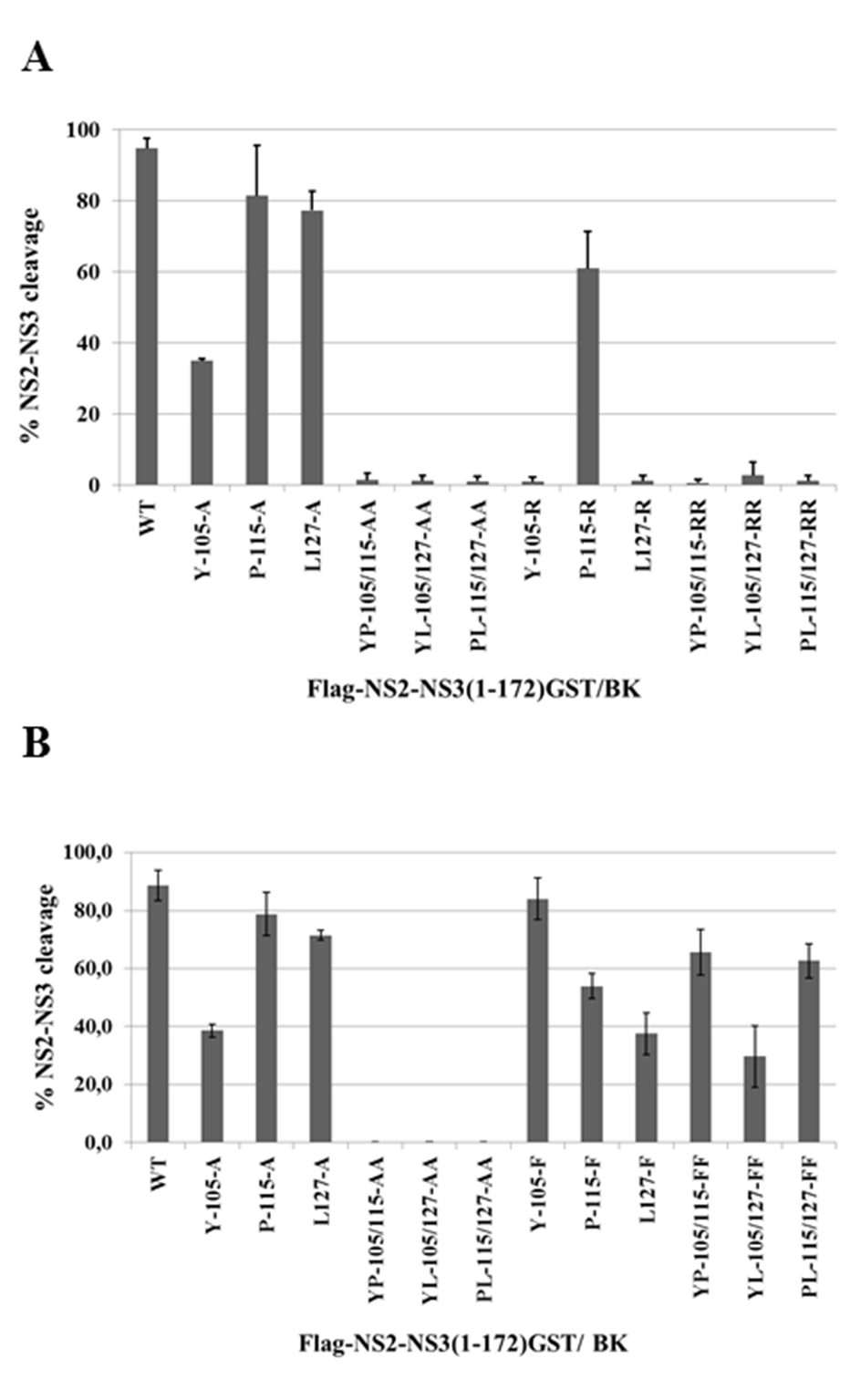

Supplement: S3 Fig — The cleavage efficiencies of the indicated BK NS2-NS3 polyprotein fragments carrying either charged (arginine shown in panel A) or hydrophobic (phenylalanine shown in panel B) NS3 amino acid substitutions were determined by Western blot analysis. Western blot signals of Flag-NS2-3(1–172)GST and Flag-NS2 of two independent Western blots were quantified by ImageJ software and the percentage of NS2-NS3 cleavage was calculated. (TIF) [file ppat.1004736.s003.tif]

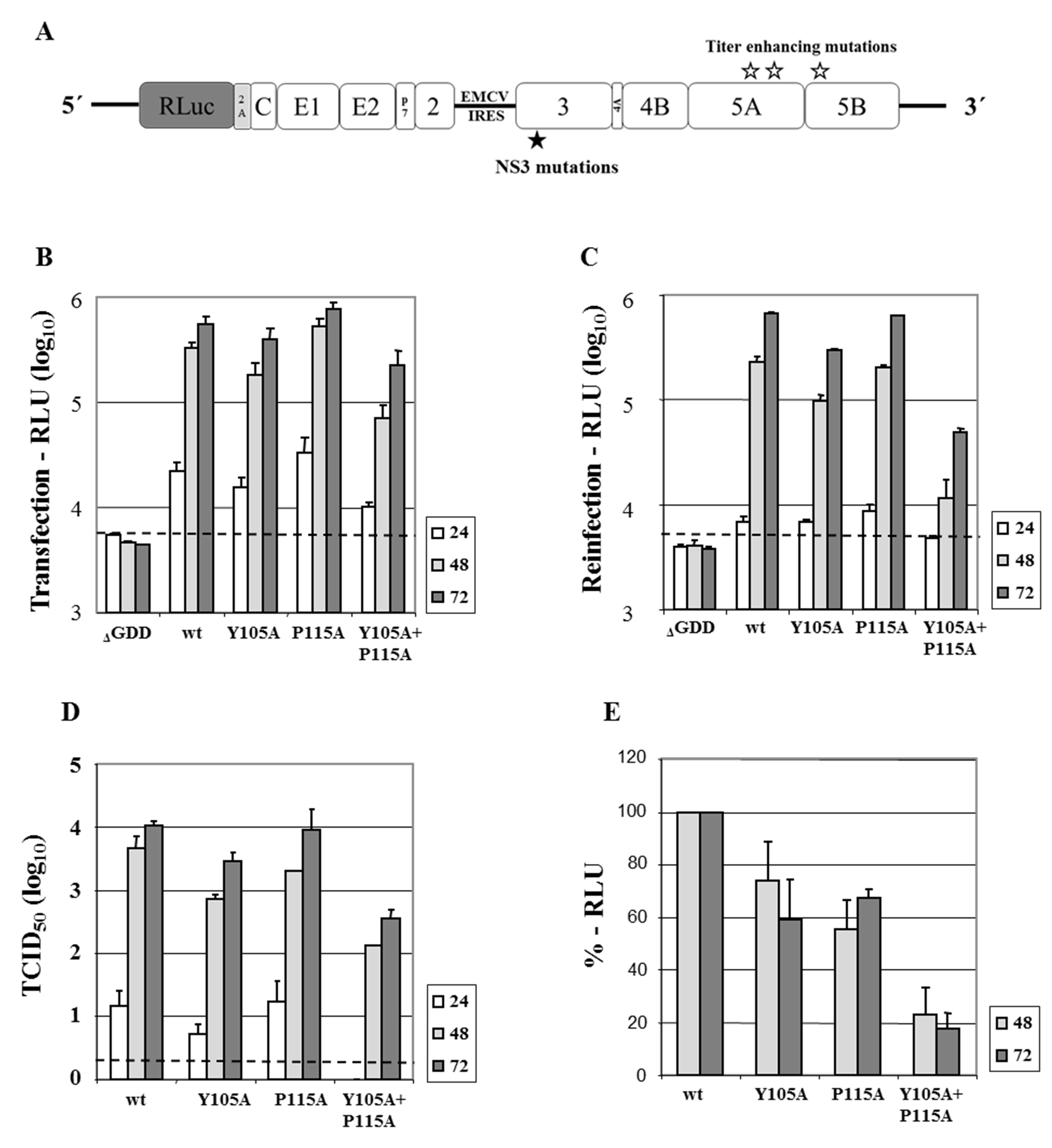

Supplement: S4 Fig — (A) Schematic diagram of the full length JFH1ad-R2a_NS2EI3 genome used for functional characterization. The HCV proteins and NTRs are represented as white boxes, renilla luciferase (RenLuc) used for indirect quantitative analysis of HCV genome is shown in dark gray. FMDV 2A peptide sequence in light gray facilitates release of authentic core protein. The EMCV IRES sequence was inserted between NS2 and NS3 sequences to separate protease activity from replication and is shown as a black box. Cell culture titer enhancing mutations in NS5A and NS5B (V2153A, V2440L and V2941M) are represented as white stars. The positions of NS3 aa substitutions Y105A and P115A are indicated as a black star. (B) Huh7.5 cells were transfected with viral RNA specified at the bottom of the graph and kinetics of HCV genome replication were quantified 24, 48 and 72 hours post transfection by luciferase assay. (C) Supernatants of transfected cells containing released infectious particles were harvested 24, 48 and 72 hours post transfection and used for infection of naïve Huh7.5 cells. After three days, infected cells were lysed and intracellular luciferase activity was measured. (D) Huh7.5 cells were transfected with RNAs specified at the bottom of the graph and release of infectious particles into culture supernatants were quantified 24, 48 and 72 hours post transfection by TCID50 assay. In panels (B), (C) and (D) representative results of two independent experiments performed in duplicates with standard deviations are shown. Dotted lines represent background values of the assays. (E) Efficiency of infectivity release was estimate as a ratio of infectivity release (luciferase value of graph C) and replication (luciferase value of graph B). (TIF) [file ppat.1004736.s004.tif]

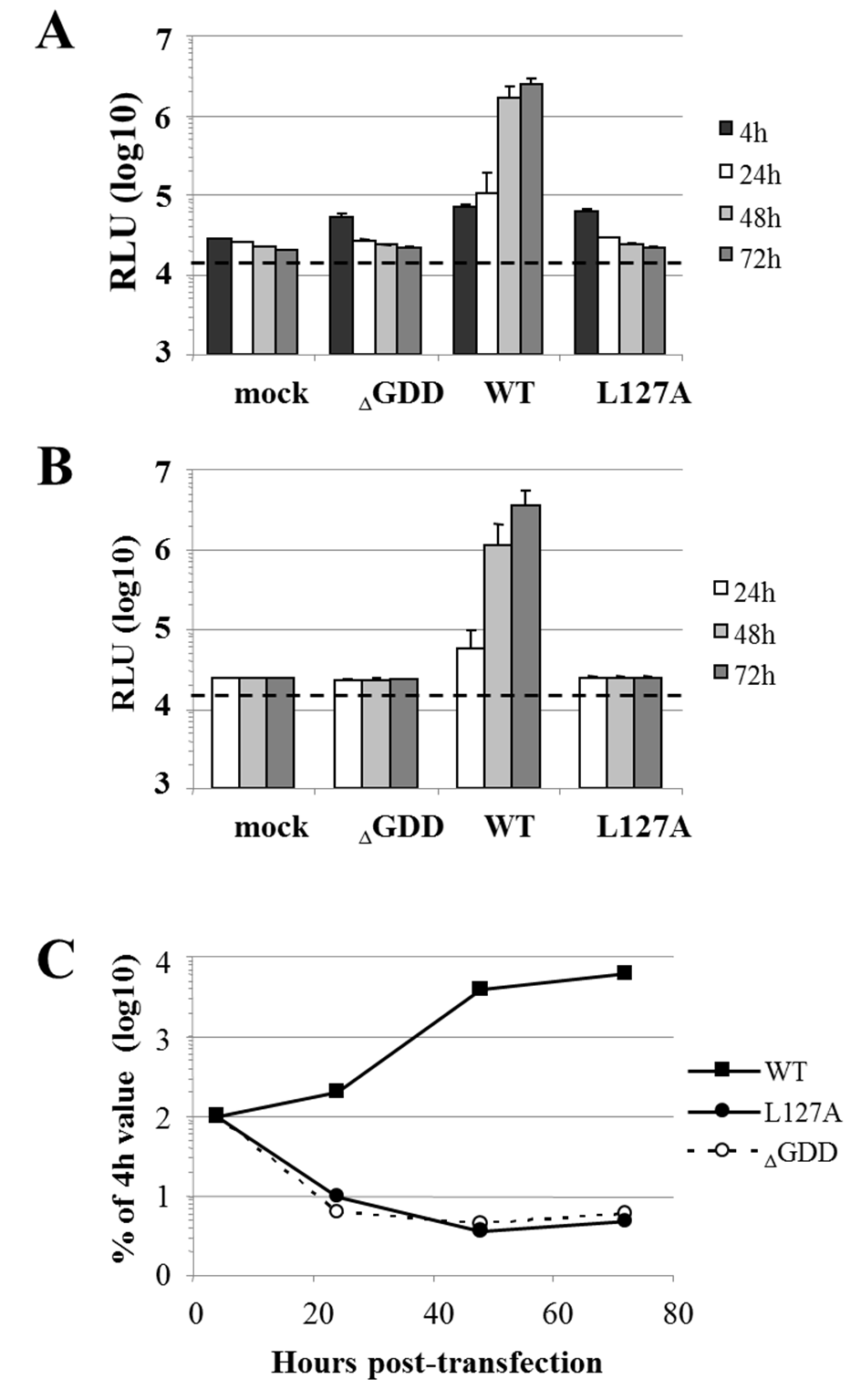

Supplement: S5 Fig — (A) Huh7.5 cells were transfected with viral RNA specified at the bottom of the graph or mock transfected and kinetics of HCV genome replication were quantified 4, 24, 48 and 72 hours post transfection by luciferase assay. (B) Supernatants of transfected cells containing released infectious particles were harvested 24, 48 and 72 hours post transfection and used for infection of naïve Huh7.5 cells. After three days, infected cells were lysed and intracellular luciferase activity was measured. (C) The kinetics of replication (data from panel A) was determined by normalizing the relative light units at the different time points to the mock value and the respective 4-h value. In panels (A) and (B) representative results of two independent experiments performed in duplicates with standard deviations are shown. Dotted lines represent background values of the assays measured with blank buffer. (TIF) [file ppat.1004736.s005.tif]

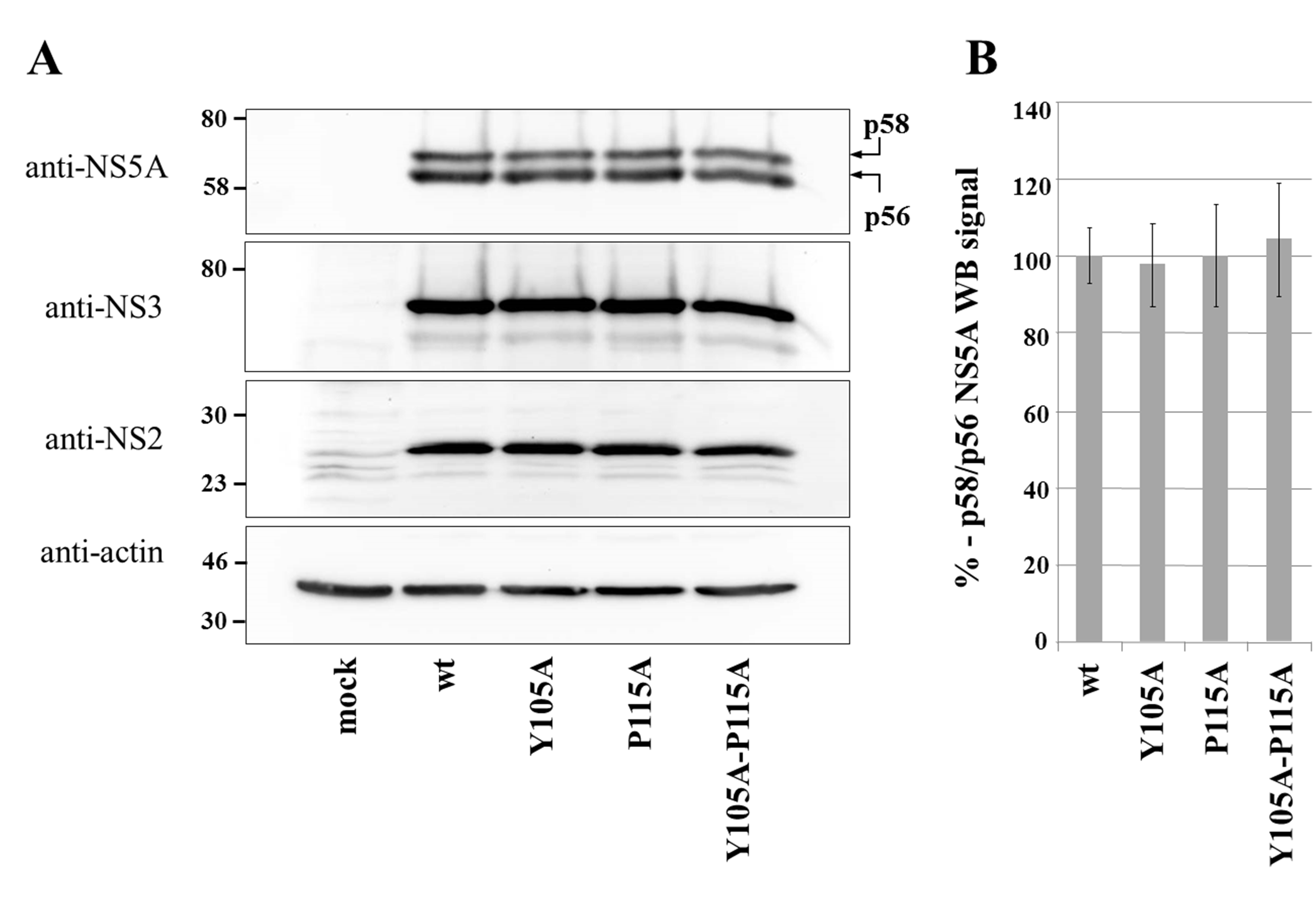

Supplement: S6 Fig — (A) Huh7.5 cells were transfected with viral RNA specified at the bottom or mock transfected, the cell lysates were harvested 72 hours post transfection and analyzed by Western Blot assay. (B) Western blot signals of NS5A p56 and p58 forms of six independent Western blots from two biological repetitions were quantified by Quantity One software and the ratio of p58 and p56 was calculated. (TIF) [file ppat.1004736.s006.tif]

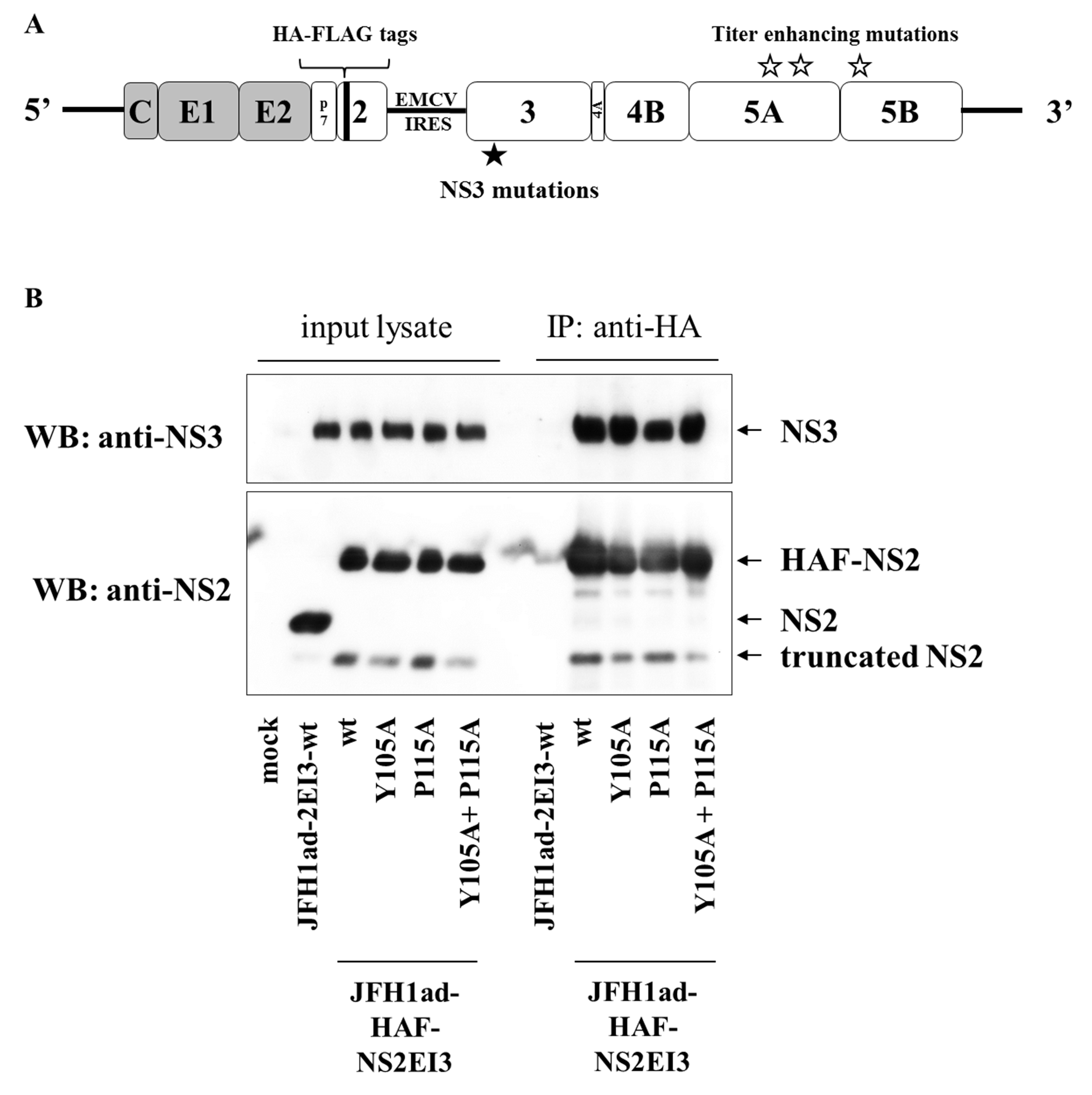

Supplement: S7 Fig — (A) A schematic diagram of the full length JFH1ad_HAF-NS2EI3 genome used for NS2/NS3 proteins co-immunoprecipitation. The HCV proteins and NTRs are represented as white boxes. EMCV IRES sequence inserted between NS2 and NS3 sequences and HA and Flag tag sequences are shown as black boxes. Cell culture titer enhancing mutations in NS5A and NS5B (V2153A, V2440L and V2941M) are represented as white stars. Position of NS3 aa substitutions Y105A and P115A is shown as a black star. (B) Huh7.5 cells were transfected with wt or genome containing an NS3 aa substitution and harvested after 72 hours and lysed. The HCV construct without HA-Flag tag sequence (JFH1ad_NS2EI3) and mock-transfected cells were used as technical negative controls. Protein samples were used for HA specific immunoprecipitation. Pull down efficiency of HAF-NS2 protein as well as co-immunoprecipitated NS3 protein were analyzed by Western Blot assay. Input lysate and sample containing immunoprecipitated proteins were loaded on the gel in the ration 1:10. (TIF) [file ppat.1004736.s007.tif]
