# Supplementary material for: A Conserved NS3 Surface Patch Orchestrates NS2 Protease Stimulation, NS5A Hyperphosphorylation and HCV Genome Replication
Source: PLoS Pathog. 2015 Mar 16;11(3):e1004736. doi: 10.1371/journal.ppat.1004736 (PMC4361677; doi:10.1371/journal.ppat.1004736)
Supplement: S1 Text — Supporting reference list. (DOC) [file ppat.1004736.s008.doc]

**Supporting Information**

**Cell culture.** Monolayers of the highly permissive cell line Huh7.5 [1] were grown in Dulbecco’s modified minimal essential medium (DMEM; Life Technologies) supplemented with 2 mM L-glutamine, nonessential amino acids, 100 U/ml of penicillin, 100 mg/ml of streptomycin, and 10% fetal calf serum.

**Plasmids.**

**For transient protein expression experiments.** The HCV BK (genotype 1b) cDNA of NS2-NS3 from pFLAG-NS2-NS3(1-172)GST/BK constructs used for the initial NS3 alanine scanning mutagenesis is the one used by Pallaoro et al. [2]. These plasmids encode the FLAG epitope, NS2 and the N-terminal 172 aa of NS3 followed by GST and were derived from pcite-FLAG-NS2-NS3(1-213)-GST [3]. The NS3-mutagenesis was directly performed on these DNAs. To generate pcite-FLAG-NS2-NS3(1-172)-GST/JFH1 PCR fragments FLAG-NS2(NotI), NS2(NotI)-NS3(1-172/AgeI) were generated by PCR amplification by using pFKI389-Luc/2-3’_JFH as template DNA. The pcite FLAG-NS2-NS3(1-172)GST/JFH was generated follows. PCR amplification of the FLAG-NS2(NotI) fragment was performed with primers FLAG-NS2 JFH1 se (5‘-ccatggactacaaggacgacgatgacaagcttgaacccgggtatgacgcacctgtgcacggacagataggcgtg-3‘) and NS2 (NotI) as (5‘-gtgacggcccacgcgatgccatcgcggccgccgcgc-3‘) and the PCR product was cloned into pGEM-T vector creating pGEM-T flag-NS2(NotI). The NS2(NotI)-NS3(1-172/AgeI) fragment was amplified with the primer pair NS2 (NotI) se (5‘-gcgcggcggccgcgatggcatcgcgtgggccgtc-3‘) and NS3(1-172-AgeI) ase (5‘-aaccggtaacggggatgaaatcgatggattt-3‘) followed by cloning of the PCR product NS2(NotI)-NS3(1-172/AgeI) in pGEM-T resulting in the generation of pGEM-T NS2(NotI)-NS3(1-172)-AgeI. The pcite FLAG-NS2-NS3(1-172)GST/JFH construct was then generated by cutting pGEM-T flag-NS2(NotI) with *NcoI*, *NotI* and pGEM-T NS2(NotI)-NS3(1-172)-AgeI with *NotI*, *AgeI* and the fragments were subsequently cloned into pcite2A vector (Novagen) cleaved with *NcoI* and *AgeI* resulting in pcite-FLAG-NS2-NS3(1-172)GST/JFH.

The JFH1 and Con1-derived expression constructs pcite-NS3-3’/JFH1, pcite-FLAG-NS2-3’/JFH1, pcite-FLAG-NS2-3’/JFH1 NS2/C-184-A (C184-A; NS2 protease active site mutation), pcite-NS3-3’/Con1, pcite-NS2-3’/Con1, pcite-NS2-3’/Con1 NS2/C-184-A (inactive NS2 protease) were all based on pcite2A (Novagen). These plasmids encode either the wild type or mutant JFH1- or Con1-derived polyprotein sequences NS3 to NS5B or NS2 to NS5B, respectively. Mutagenesis of genotype 1b (BK) NS3 was performed on either pcite-FLAG-NS2-NS3(1-172)GST or pcite-NS3(1-172)GST plasmids. QuikChange mutagenesis of Con1 NS3 was performed on pBluescript KS(+) containing a HindIII-XhoI fragment (pKS-HindIII-XhoI/Con1) of the Con1 cDNA derived from pFK-I_341_PI-Luc/NS3-3′/Con1/ET. Site-directed mutagenesis of JFH1 NS3 was performed on a pLITMUS28i (NEB) derivate containing a KpnI-NsiI JFH1 cDNA fragment (plit-KpnI-NsiI/NS3/JFH1) from pFKI389Luc/NS3-3’_dg. Verified mutations were cloned into either pcite-NS3-NS5B/JFH1 or pFKI389Luc/NS3-3’_dg via KpnI/NsiI sites. Mutagenesis of JFH1 NS2-NS3 cDNA was performed on a plit28-KpnI-NsiI/NS2-NS3/JFH1 subclone containing the NS2-NS3/JFH1 fragment until the NsiI site from pFKI389Luc/NS2-3’_dg. The internal KpnI site has been destroyed by a silent mutation with oligos NS2JFH KpnI QC se (5’-gaagccatgattcaggagtgggtcccacccatgcaggtgcgcg-3’) NS2JFH KpnI QC ase (5’-cgcgcacctgcatgggtgggacccactcctgaatcatggcttc-3’). All mutations were cloned into either pcite-NS2-NS5B/JFH1 or pFKI389Luc/NS2-3’_dg via KpnI/NsiI sites. The pcite-FLAG-MBP-NS4B-NS5A-trx-HA encodes the NS3-4A serine protease substrate FLAG-MBP-NS4B-NS5A-trx-HA downstream of the EMCV internal ribosomal entry site. The minimal NS3-4A substrate consists of the last 20 aa of NS4B followed by the first 20aa of NS5A- representing the minimal NS4B-5A cleavage site of genotype 1b (BK)- flanked at the N-terminus by a FLAG-MBP (maltose binding protein) tag and C-terminally fused to a thioredoxin-HA tag. The pcite-FLAG-MBP-NS4B-NS5A-trx-HA encodes these protein-coding sequences downstream of the internal ribosomal entry site. The pcite-V5-NS4A/BK codes downstream of the IRES sequence for the NS4A gene of HCV genotype 1b/BK fused to V5 epitope tag.

**For replication studies with subgenomic HCV replicons.** The subgenomic genotype 2a (JFH1) replicon constructs pFKI389Luc/NS3-3’_dg (designated pFKI389-Luc/3-3’_JFH), pFKI389Luc/NS2-3’_dg (abbreviated pFKI389-Luc/2-3’_JFH) and the replication-deficient mutant versions FKI389-Luc/3-3’_JFH/GND and FKI389-Luc/2-3’_JFH/GND have been described [4]. The subgenomic genotype 1b (Con1) replicons pFK-I_341_PI-Luc/NS3-3′/Con1/ET (designated FKI341-Luc/3-3’_Con1), pFK-I_341_PI-Luc/NS3-3′/Con1/GND and the NS2-5B derivatives pFK-I_341_PI-Luc/NS2-3′/Con1/ET (designated FKI341-Luc/2-3’_Con1), pFK-I_341_PI-Luc/NS2-3′/Con1/GND (designated Luc-Con1/2-5B/GND) were described elsewhere [5,6].

**For replication, infectivity release and co-immunoprecipitation assays using full length bicistronic HCV genomes.** All nucleotide and aa numbers refer to the JFH1 genome (GenBankTM accession number AB047639). JFH1ad_NS2EI3, JFH1ad_HAF-NS2EI3 and JFH1ad-R2a_NS2EI3 were derived from JFH1 sequence [7] but containing three virus titer enhancing mutations (V2153A, V2440L and V2941M) [8]. NS2 and NS3 genes are separated by internal ribosome entry site (IRES) of the encephalomyocarditis virus (EMCV) [9].

In the JFH1ad-R2a_NS2EI3 construct, the gene encoding the renilla luciferase (RenLuc) was fused N-terminally with the 16 N-terminal aa of the core protein and C-terminally with the foot-and-mouth disease virus (FMDV) 2A peptide coding region [10]. JFH1ad _HAF-NS2EI3 contains HA and Flag tag sequence inserted in frame into the NS2 N-terminus [11].

NS3 Y105A, P115A and YP105/115AA mutations were transferred into the JFH1 full length constructs from plit28-NS2-IRES-NS3/JFH1 plasmids via NotI and NsiI restriction sites.

**Co-immunoprecipitation.** Huh7.5 cells were mock treated or transfected with HCV RNA, and samples were harvested 72 h later by scraping into IP buffer (0.5% n-dodecyl-ß-D-maltoside, 100 mM NaCl, 20 mM Tris pH 7,5). After 60 min incubation on ice, cell debris was removed by 45 min centrifugation at 20,000g. Samples containing soluble proteins were incubated with HA-specific antibody beads (Sigma Aldrich) four hours at 4°C. After five times washing with IP buffer, samples were eluted into sample buffer and separated by SDS-PAGE gel. Proteins were transferred onto PVDF membrane and HCV proteins were detected by rabbit polyclonal antibodies.

**Immunohistochemical staining and virus titration.** Virus titers were determined as described elsewhere with slight modifications [12]. In brief, Huh7.5 cells were seeded into 96-well plates and fixed 3 days after infection. For immunohistochemistry we used an antibody specific for the JFH1 NS3 helicase (2E3, generated in cooperation with H. Tang, Florida State University, USA) at a dilution of 1:500. Bound antibody was detected with a peroxidase-conjugated secondary antibody specific to murine IgG (Sigma-Aldrich) diluted 1:200 in PBS. Virus titers (50% tissue culture infective dose per ml; [TCID50/ml]) were calculated as described previously [13].

**Luciferase assays using full length bicistronic HCV genomes.** Quantification of luciferase reporter activity was used to determine HCV RNA replication and assembly as described previously [13] with some modifications. In brief, HCV transfected Huh7.5 cells were seeded in duplicate into 6-well plates. The supernatants of transfected cells were harvested 1, 2 and 3 days after transfection and used for reinfection of a naïve cells. Transfected cells were lysed in 350 µl of lysis buffer (0.1% Triton X-100, 25 mM glycylglycine, 15 mM MgSO_4_, 4 mM EGTA) 1, 2 or 3 days after transfection. Reinfected Huh7.5 cell were lysed in the lysis buffer 3 days after infection. Intracellular luciferase counts of transfected cells represent viral replication while values of reinfected cells represent infectivity release of transfected HCV genomes. For each well, 20µl of lysate was mixed with 100µl of luciferase assay buffer (15mM K-phosphat, pH7,8; 25mM GlycylGlycine, pH7,8; 15mM MgSO4; 4 mM EGTA, Coelenterazine 117µM) and measured in a luminometer (Lumat LB9507; Berthold). Each sample was measured in duplicate.

**Antisera.** The following antisera were used: rabbit polyclonal antibody specific for NS2 (NS2-1519) [14]; rabbit polyclonal antibody specific for NS3 of JFH1 (NS3-4949) [5]; mouse monoclonal antibody specific for JFH1 NS3 (NS3-2E3; generated in co-operation with H. Tang, Florida State University, USA).

**Production of mAbs anti-NS3 (4D11) and anti-NS2 (YAL-4-70-8):** BALB/c mice were primed and boosted at 3-week intervals with recombinant HCV NS3 protein (25 micrograms) that was complexed with adjuvant (RIBI Immunochemical). Approximately 1 month after the last boost, we harvested serum and tested it for immunoreactivity against HCV-infected Huh-7.5 cells. Mice with high titers (>1/1,000) were boosted intravenously with purified NS3 protein (15 micrograms) in PBS. We harvested splenocytes 3 d later and fused them to P3X63Ag8.653 myeloma cells to generate hybridomas according to published procedures (REF below). We purified monoclonal antibodies against by standard protein A or protein G chromatography.

Rabbit monoclonal antibody specific for NS2 (YAL-4-70-8) was created in collaboration with Epitomics, Inc. (Burlingame, CA) under approval from Yale University Institutional Animal Care and Use Committee. Briefly, New Zealand White rabbits were immunized with complete Freund’s adjuvant and keyhole limpet hemocyanin (KLH) conjugated to NS2- specific peptide TPMSDWAASGLRDLAV. Following four additional boosts with the KLH-conjugated peptide plus incomplete Freund’s adjuvant, animals were primarily screened via ELISA for reactivity to the relevant peptide conjugated to bovine serum albumin and secondarily by western blot to Jc1-infected Huh-7.5 cell lysates. Animals exhibiting strong peptide-specific responses were sacrificed by intramuscular injection with 60 mg/Kg ketamine plus 5mg/Kg xylazine, followed by intravenous injection of euthasol 22ml/Kg. Following splenectomy, lymphocytes isolated and fused to a proprietary rabbit hybridoma partner (Epitomics). Reactive hybridomas were cloned by limiting dilution, screened as above, and subcloned two additional times. Purified monoclonal antibodies were produced from the hybridomas by Cell Essentials (Boston, MA).

**Supporting References list**

1. Blight KJ, McKeating JA, Marcotrigiano J, Rice CM (2003) Efficient Replication of Hepatitis C Virus Genotype 1a RNAs in Cell Culture. J Virol 77: 3181–3190.

2. Pallaoro M, Lahm A, Biasiol G, Brunetti M, Nardella C, et al. (2001) Characterization of the hepatitis C virus NS2/3 processing reaction by using a purified precursor protein. J Virol 75: 9939–9946.

3. Schregel V, Jacobi S, Penin F, Tautz N (2009) Hepatitis C virus NS2 is a protease stimulated by cofactor domains in NS3. Proc Natl Acad Sci U S A 106: 5342–5347. doi:0810950106 [pii] 10.1073/pnas.0810950106.

4. Schaller T, Appel N, Koutsoudakis G, Kallis S, Lohmann V, et al. (2007) Analysis of hepatitis C virus superinfection exclusion by using novel fluorochrome gene-tagged viral genomes. J Virol 81: 4591–4603. doi:JVI.02144-06 [pii] 10.1128/JVI.02144-06.

5. Lohmann V, Hoffmann S, Herian U, Penin F, Bartenschlager R (2003) Viral and cellular determinants of hepatitis C virus RNA replication in cell culture. J Virol 77: 3007–3019.

6. Windisch MP, Frese M, Kaul A, Trippler M, Lohmann V, et al. (2005) Dissecting the interferon-induced inhibition of hepatitis C virus replication by using a novel host cell line. J Virol 79: 13778–13793. doi:79/21/13778 [pii] 10.1128/JVI.79.21.13778-13793.2005.

7. Wakita T, Pietschmann T, Kato T, Date T, Miyamoto M, et al. (2005) Production of infectious hepatitis C virus in tissue culture from a cloned viral genome. Nat Med 11: 791–796. doi:10.1038/nm1268.

8. Kaul A, Woerz I, Meuleman P, Leroux-Roels G, Bartenschlager R (2007) Cell culture adaptation of hepatitis C virus and in vivo viability of an adapted variant. J Virol 81: 13168–13179. doi:JVI.01362-07 [pii] 10.1128/JVI.01362-07.

9. Jirasko V, Montserret R, Appel N, Janvier A, Eustachi L, et al. (2008) Structural and functional characterization of nonstructural protein 2 for its role in hepatitis C virus assembly. J Biol Chem 283: 28546–28562. doi:M803981200 [pii] 10.1074/jbc.M803981200.

10. Reiss S, Harak C, Romero-Brey I, Radujkovic D, Klein R, et al. (2013) The lipid kinase phosphatidylinositol-4 kinase III alpha regulates the phosphorylation status of hepatitis C virus NS5A. PLoS Pathog 9: e1003359. doi:10.1371/journal.ppat.1003359.

11. Jirasko V, Montserret R, Lee JY, Gouttenoire J, Moradpour D, et al. (2010) Structural and functional studies of nonstructural protein 2 of the hepatitis C virus reveal its key role as organizer of virion assembly. PLoS Pathog 6: e1001233. doi:10.1371/journal.ppat.1001233.

12. Lindenbach BD, Evans MJ, Syder AJ, Wölk B, Tellinghuisen TL, et al. (2005) Complete replication of hepatitis C virus in cell culture. Science 309: 623–626. doi:10.1126/science.1114016.

13. Koutsoudakis G, Herrmann E, Kallis S, Bartenschlager R, Pietschmann T (2007) The level of CD81 cell surface expression is a key determinant for productive entry of hepatitis C virus into host cells. J Virol 81: 588–598. doi:JVI.01534-06 [pii] 10.1128/JVI.01534-06.

14. Jirasko V, Montserret R, Appel N, Janvier A, Eustachi L, et al. (2008) Structural and functional characterization of nonstructural protein 2 for its role in hepatitis C virus assembly. J Biol Chem 283: 28546–28562. doi:M803981200 [pii] 10.1074/jbc.M803981200.
